# CYP2D6 as an Emerging Endogenous Oxidative Stress Modulator in Cardiovascular Disease: Genetic, Pharmacological, and Redox Perspectives

**DOI:** 10.3390/antiox15091154

**Published:** 2026-09-10

**Authors:** Cheng-Wu Yang, Wen-Hua Chen, Tzong-Shyuan Lee

**Affiliations:** Graduate Institute and Department of Physiology, College of Medicine, National Taiwan University, Taipei 10051, Taiwan; r12441007@ntu.edu.tw (C.-W.Y.); f08441005@ntu.edu.tw (W.-H.C.)

**Keywords:** CYP2D6, cardiovascular disease, oxidative stress, reactive oxygen species, pharmacogenomics, phenoconversion, precision cardiology, polypharmacy

## Abstract

Oxidative stress is a central and well-established driver of cardiovascular disease, contributing to mitochondrial dysfunction, endothelial injury, inflammatory activation, and progressive myocardial and vascular remodeling. Although the major endogenous sources of cardiovascular reactive oxygen species (ROS), including NADPH oxidases, mitochondrial electron transport chain leakage, and uncoupled nitric oxide synthase, are well characterized, an additional and underappreciated contributor has recently emerged: cytochrome P450 2D6 (CYP2D6), an enzyme classically regarded as a hepatic drug-metabolizing protein. Accumulating evidence indicates that CYP2D6 is expressed extrahepatically in cardiac, vascular, and neural tissue, where uncoupled catalytic cycling is proposed to generate ROS independently of its canonical xenobiotic-metabolizing role, although direct experimental evidence for this pathway in human cardiac and vascular tissue remains limited. CYP2D6-derived oxidative processes may interact with mitochondrial respiratory function, endothelial nitric oxide bioavailability, and redox-sensitive inflammatory pathways, potentially contributing to cardiovascular vulnerability under specific genetic or pathological conditions. Critically, the magnitude of this oxidative contribution is not fixed: it is dynamically shaped by inherited CYP2D6 genetic variation, with poor and ultra-rapid metabolizer phenotypes exhibiting divergent oxidative burden and pharmacokinetic vulnerability, and is further amplified by polypharmacy, multimorbidity, and inflammation-driven phenoconversion, whereby clinically expressed CYP2D6 activity diverges from inherited genotype in ways that intensify redox imbalance. These dynamics are particularly relevant in East Asian populations, where the decreased-function CYP2D6*10 allele is highly prevalent. In this narrative, hypothesis-generating review, we integrate evidence from pharmacogenomics, redox biology, and cardiovascular pharmacology to propose a conceptual framework that reframes CYP2D6 as a genetically and pharmacologically tunable node within cardiovascular redox biology. We further examine emerging redox biomarkers, multi-omics platforms, and AI-assisted modeling as translational strategies for capturing this dynamic oxidative risk in real time. This framework supports a shift from static genotype-guided prescribing toward oxidative-risk-informed, adaptive cardiovascular precision medicine. Importantly, our focus on CYP2D6 should not be interpreted as evidence that it is a major cardiovascular CYP isozyme or an established driver of cardiovascular pathology. Rather, CYP2D6 is examined here as a deliberately hypothesis-generating candidate whose unusually strong pharmacogenetic variability, clinically important cardiovascular drug substrates, dynamic susceptibility to phenoconversion, extrahepatic expression, and mechanistically plausible links to endogenous substrate metabolism and CYP-associated ROS generation provide a convergent rationale for focused investigation. The mechanistic framework proposed in this review has not yet been experimentally and prospectively validated and should not be applied directly to clinical decision-making without supporting clinical data.

## 1. Introduction

Literature Search Methodology: As a narrative, hypothesis-generating review, this manuscript is based on a non-systematic search of the PubMed/MEDLINE and Scopus databases for English-language articles from database inception through July 2026, using combinations of the terms “CYP2D6,” “cardiovascular,” “oxidative stress,” “reactive oxygen species,” “phenoconversion,” “pharmacogenomics,” “cytochrome P450,” and related terms, supplemented by manual screening of the reference lists of retrieved articles and relevant prior reviews. Source selection prioritized studies directly addressing CYP2D6 biology; where CYP2D6-specific data were unavailable, context was drawn from the broader cytochrome P450 literature, and this is noted explicitly at the relevant points in the text. This search strategy is disclosed here to enable readers to appropriately weigh the evidentiary basis for specific claims throughout the review.

### 1.1. Cardiovascular Disease Burden and the Need for Precision Cardiology

Cardiovascular disease (CVD) remains the leading cause of mortality worldwide despite substantial advances in pharmacological and interventional therapies [1]. Nevertheless, considerable variability in therapeutic response and long-term clinical outcomes continues to limit the effectiveness of current cardiovascular management [2]. Conventional treatment paradigms cannot fully account for this variability, underscoring the need for more individualized therapeutic strategies. Precision cardiology has therefore emerged as a promising clinical paradigm that integrates genetic, molecular, and clinical information to optimize patient-specific therapeutic decision-making [3,4,5,6,7].

Despite these advances, individualized cardiovascular therapy remains challenging because drug response is influenced by far more than inherited genetic variation alone [4]. However, aging, comorbidities, organ dysfunction, environmental exposures, and polypharmacy may collectively modify drug disposition and therapeutic response, contributing to interindividual variability in cardiovascular treatment outcomes [5,6]. Consequently, although precision cardiology has substantially advanced individualized prescribing, considerable variability in therapeutic response and cardiovascular outcomes remains unexplained, supporting the need for precision medicine models that integrate both inherited and acquired determinants of drug response [7].

Current pharmacogenomic frameworks explain only part of the observed variability in cardiovascular drug response. Although genotype-guided prescribing has substantially advanced individualized therapy, considerable discrepancies remain between genetically predicted metabolic phenotypes and real-world therapeutic outcomes. These observations highlight an important conceptual transition from static genotype-based prediction toward a dynamic model incorporating physiological, pathological, and environmental modifiers that evolve over time. Among the pharmacogenes implicated in cardiovascular medicine, CYP2D6 represents an informative paradigm for investigating these interactions because of its extensive genetic polymorphism and marked interindividual variability in predicted metabolic activity [8]. Beyond these pharmacogenomic functions, accumulating evidence suggests that CYP2D6 may participate in biological processes extending beyond hepatic xenobiotic metabolism, including the metabolism of endogenous neuroactive substrates [9]. These observations suggest that CYP2D6 may indirectly influence neurohumoral and cardiovascular biology, although direct mechanistic evidence remains limited. Although these observations remain fragmented across pharmacogenomic, mechanistic, and clinical studies, they collectively suggest that CYP2D6 may play a broader role in cardiovascular biology than previously appreciated. Integrating these complementary lines of evidence provides the rationale for re-examining CYP2D6 within a systems-level cardiovascular framework, which forms the conceptual basis for the three-layer model proposed throughout this review.

### 1.2. Current Limitations of CYP2D6 Pharmacogenomics in Cardiovascular Medicine

Among currently available pharmacogenetic biomarkers, CYP2D6 has become one of the most clinically relevant determinants of interindividual variability in cardiovascular pharmacotherapy because it contributes to the biotransformation of approximately 20–25% of clinically prescribed drugs, including numerous cardiovascular and centrally acting agents [10]. This prominent role in drug metabolism has established CYP2D6 as a cornerstone of contemporary pharmacogenomic-guided therapy. Genetically, CYP2D6 is located on chromosome 22q13.2 and encodes a highly polymorphic monooxygenase belonging to the cytochrome P450 superfamily [11]. To date, more than 170 star (*) alleles and numerous subvariants have been identified, arising from single-nucleotide polymorphisms, gene deletions, duplications, hybrid rearrangements, and copy number variations that collectively generate substantial interindividual variability in enzymatic activity [12]. Based on allele combinations and predicted enzymatic function, individuals are conventionally classified into four metabolizer phenotypes: poor metabolizers (PMs), intermediate metabolizers (IMs), normal metabolizers (NMs), and ultra-rapid metabolizers (UMs) [13]. PMs typically carry two nonfunctional alleles and exhibit markedly diminished or absent CYP2D6 activity, whereas UMs usually harbor gene duplications or multiplications that confer accelerated drug metabolism and clearance [14]. The distribution of CYP2D6 variants differs markedly among ethnic populations, with the decreased-function CYP2D6*10 allele occurring in approximately 35–60% of East Asian individuals [15], with country-level estimates ranging from approximately 36% in Japan to approximately 46% in Korea. In contrast, functional gene duplications are more frequently observed in Middle Eastern and North African populations [16]. These population-specific differences further highlight the clinical relevance of CYP2D6 pharmacogenomics for individualized cardiovascular pharmacotherapy across diverse ancestral backgrounds.

The clinical relevance of CYP2D6 pharmacogenomics in cardiology is well established and best illustrated by its influence on cardiovascular drug exposure and response. Metoprolol represents a prototypical substrate: reduced CYP2D6 activity is associated with enhanced β-blocker effects, including lower heart rate and blood pressure [17]. Similarly, antiarrhythmic agents such as flecainide and propafenone are strongly CYP2D6-dependent, and aberrant metabolism may predispose patients to pro-arrhythmic toxicity and sudden cardiac events [18]. In recognition of clinically actionable pharmacogenetic relationships, CPIC and DPWG have developed standardized frameworks for translating pharmacogenetic results into clinical prescribing guidance [19,20].

Despite the demonstrated utility of genotype-guided prescribing, meaningful discrepancies frequently arise between predicted CYP2D6 phenotypes and observed drug responses in real-world cardiovascular practice [21]. Collectively, these observations suggest that conventional CYP2D6 pharmacogenomics provides an essential foundation for individualized therapy but does not fully explain the dynamic variability observed in cardiovascular drug response. These limitations support a transition from static genotype-based prediction toward a broader systems-level understanding of CYP2D6 biology.

### 1.3. Beyond the Genotype: Phenoconversion, Inflammatory Modulation, and Dynamic CYP2D6 Activity

One of the most important manifestations of this systems-level perspective is phenoconversion, a process in which disease states, inflammation, concomitant medications, and environmental influences independent of inherited genotype dynamically modify CYP2D6 activity. Consequently, although the inherited CYP2D6 genotype remains a major determinant of metabolic capacity, it often cannot predict clinical phenotype and therapeutic outcomes [22,23,24]. Phenoconversion provides a mechanistic explanation for this genotype–phenotype discordance. Increasing evidence indicates that CYP2D6 activity can be substantially modified by environmental, pathological, and pharmacological factors, leading to considerable temporal variability in enzyme function [23]. This phenomenon has contributed to the emerging concept of “phenotype instability,” in which an individual’s actual metabolic phenotype deviates from genetically predicted activity [23,24,25]. Potent CYP2D6 inhibitors can substantially reduce functional CYP2D6 activity and produce clinically important phenoconversion [26]. Importantly, this issue is particularly relevant to patients with cardiovascular disease because of the high prevalence of polypharmacy, multimorbidity, and concomitant neuropsychiatric pharmacotherapy [27].

Beyond drug-mediated inhibition, chronic inflammation and oxidative stress are increasingly recognized as important modulators of hepatic cytochrome P450 expression and activity [28]. Pro-inflammatory cytokines such as interleukin-6 (IL-6), tumor necrosis factor-α (TNF-α), and interferon-γ can suppress hepatic CYP expression through transcriptional and post-transcriptional mechanisms [29]. It should be noted that this cytokine-mediated suppression has been most extensively documented for CYP3A4, CYP1A2, and CYP2C subfamily enzymes. CYP2D6 is classically among the less inducible and comparatively inflammation-resistant of the major CYPs, and the magnitude of clinically significant CYP2D6 phenoconversion attributable specifically to inflammation, as distinct from inhibitor-driven phenoconversion, remains debated.

Disease-related physiological changes, particularly those accompanying heart failure, may further modify drug disposition independently of inherited genotype [30]. Moreover, oxidative stress-induced mitochondrial dysfunction and redox imbalance may further amplify cardiovascular injury, providing a plausible biological context in which altered CYP activity could interact with disease progression [31].

These observations collectively challenge the traditional paradigm of static pharmacogenetics and support a transition toward dynamic precision medicine models that integrate genomic information with real-time physiological, inflammatory, and environmental variables [32]. Rather than functioning solely as a genetically predetermined drug-metabolizing enzyme, CYP2D6 should instead be viewed as a highly adaptable systems-level regulator influenced by both intrinsic and extrinsic factors.

Rationale for focusing on CYP2D6. CYP2D6 is a comparatively minor CYP isozyme in the cardiovascular system, and we do not propose that it is a dominant cardiac CYP or an established major source of cardiovascular ROS. The present focus is intentionally speculative and hypothesis-generating. Nevertheless, several independent observations provide an evidence-driven basis for asking whether CYP2D6 could be disproportionately relevant despite its low cardiovascular abundance. First, CYP2D6 is among the most polymorphic clinically actionable pharmacogenes, producing large interindividual and ancestry-associated differences in functional activity [12,13,14,15,16]. Second, it directly determines exposure to several cardiovascular drugs with clinically important concentration-dependent effects, including metoprolol, flecainide, and propafenone [17,18]. Third, functional CYP2D6 activity is not determined by genotype alone but can be markedly altered by potent inhibitors and polypharmacy, creating phenoconversion and drug–drug–gene interactions that are especially relevant in multimorbid cardiovascular patients [21,22,23,24,25,26,27]. Fourth, CYP2D6 is expressed outside the liver, including in the brain and at low levels in cardiac tissue, and has been implicated in the metabolism of selected endogenous neuroactive substrates, providing a plausible—although unproven—link to neurohumoral cardiovascular regulation [9,33,34,35,36,37,38,39,40,41,42,43,44,45,46,47]. Fifth, CYP catalytic uncoupling is a recognized mechanism of ROS generation [48,49,50,51]; whether CYP2D6 contributes meaningfully to cardiovascular ROS remains unclear, but this chemistry provides a testable mechanistic bridge between CYP2D6 activity and redox biology. Thus, CYP2D6 is highlighted not because current evidence establishes it as a key cardiovascular pathophysiological enzyme, but because these pharmacogenetic, pharmacological, extrahepatic, and mechanistic lines of evidence converge on a coherent set of experimentally testable hypotheses. This review therefore organizes these otherwise fragmented observations, distinguishes established evidence from extrapolation, and defines the studies needed to determine whether CYP2D6’s proposed cardiovascular role is biologically and clinically meaningful.

### 1.4. A Dual-Role Framework: CYP2D6 as a Candidate Systems-Level Modifier in Cardiovascular Pharmacology and Redox Biology

Emerging observations suggest that CYP2D6 may have biological relevance beyond its established role as a pharmacokinetic determinant, although its direct contribution to cardiovascular pathophysiology remains unproven. In this review, we therefore propose a dual-role, hypothesis-generating framework that places CYP2D6 at the intersection of endogenous redox biology and exogenous pharmacologic perturbation. This framework is intended to organize testable relationships among genetic variation, functional enzyme activity, pharmacological exposure, and cardiovascular vulnerability rather than to identify CYP2D6 as a quantitatively dominant cardiovascular enzyme. CYP2D6 remains a comparatively minor cardiac CYP isoform relative to CYP2J2, and its proposed redox contribution requires direct comparison with established cardiovascular ROS sources.

The first layer is defined by genetic architecture, the inherited constellation of CYP2D6 polymorphisms, structural variants, copy-number variations, and ethnicity-specific allelic distributions that establish an individual’s baseline metabolic capacity [12]. These genomic determinants set the foundational parameters of cardiovascular pharmacokinetics and define the spectrum of susceptibility to drug accumulation, therapeutic failure, and genotype-driven adverse outcomes.

The second layer encompasses redox and pathophysiologic modulation. Beyond their canonical role in drug biotransformation, cytochrome P450 enzymes can generate ROS during uncoupled catalytic cycling; by extension, CYP2D6 may contribute to oxidative stress under conditions that favor inefficient catalytic coupling [52]. If generated locally, such oxidative stress could interact with established redox-sensitive processes involved in endothelial dysfunction, inflammation, and cardiovascular remodeling [53]. Conversely, cardiovascular disease-associated inflammation and metabolic stress may suppress hepatic CYP activity through cytokine-mediated regulatory mechanisms, potentially contributing to genotype–phenotype discordance [28,29,54].

The third layer concerns polypharmacy-driven phenoconversion, in which concurrent medications, environmental exposures, and systemic inflammatory states may dynamically perturb CYP activity, contributing to genotype–phenotype discordance [55]. Drug–drug–gene interactions involving CYP2D6 substrates and inhibitors can lead to clinically meaningful pharmacokinetic effects and contribute to therapeutic unpredictability [56]. These interactions carry particular clinical weight in aging populations bearing the compounded burden of multimorbidity and extensive polypharmacy.

**Figure 1:**
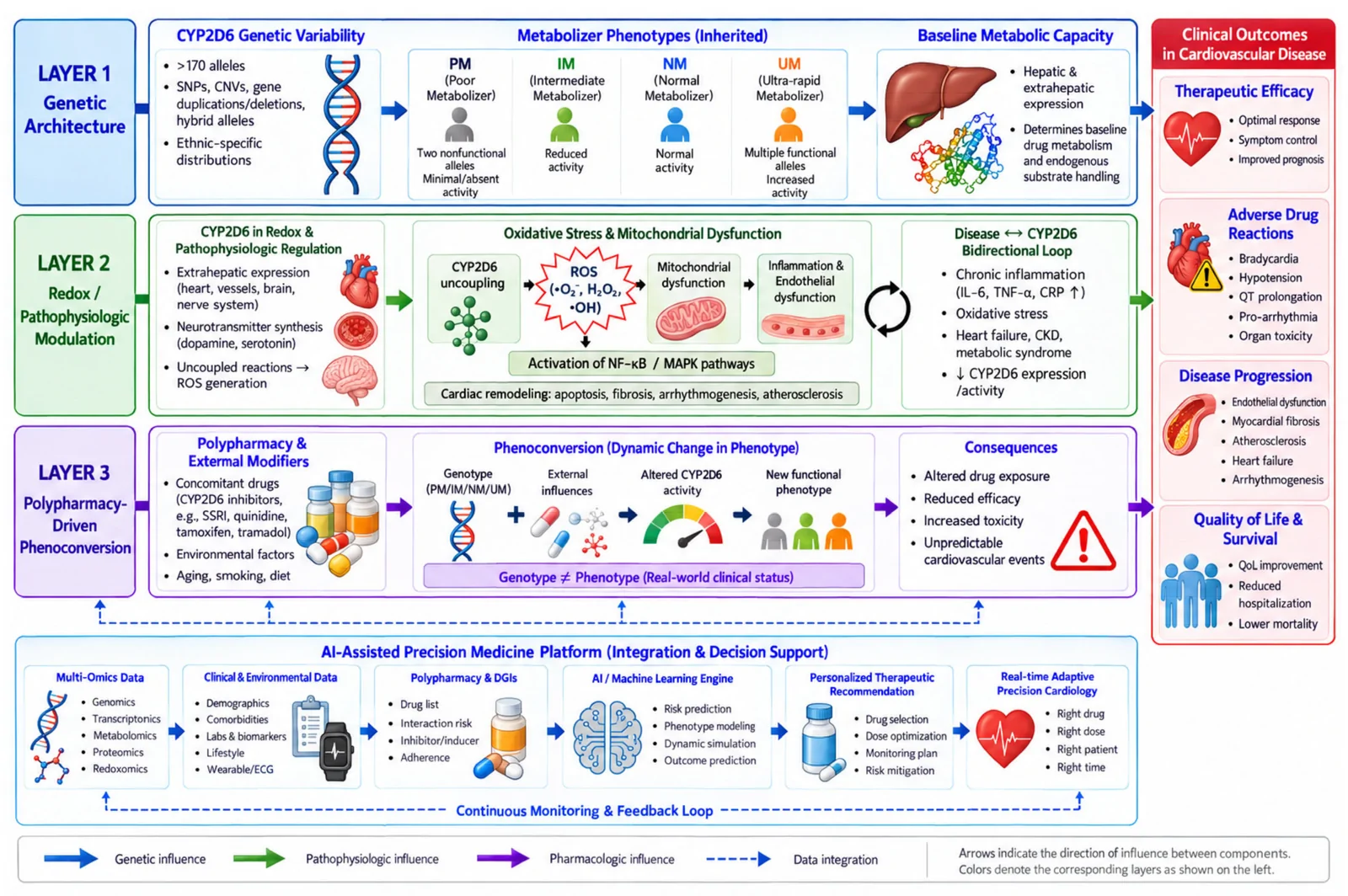
The three-layer systems-biology framework for CYP2D6 in cardiovascular disease. The framework integrates genetic, pathophysiological, and environmental/pharmacological determinants of CYP2D6 activity and their contributions to cardiovascular outcomes. Layer 1 (Genetic Architecture) illustrates how CYP2D6 genetic variability, including single-nucleotide polymorphisms, copy-number variations, gene duplications/deletions, hybrid alleles, and population-specific allele distributions, determines inherited metabolizer phenotypes and baseline metabolic capacity. Layer 2 (Redox/Pathophysiologic Modulation) depicts the potential involvement of CYP2D6-related metabolic activity in oxidative stress and mitochondrial dysfunction, with downstream activation of inflammatory and stress-responsive pathways that may contribute to endothelial dysfunction, cardiomyocyte injury, cardiac remodeling, and atherosclerosis. Conversely, cardiovascular and systemic disease states may alter CYP2D6 activity through inflammation and oxidative stress, creating a bidirectional relationship between disease and drug-metabolizing capacity. Layer 3 (Polypharmacy-Driven Phenoconversion) highlights how concomitant medications, environmental exposures, aging, smoking, diet, and other external modifiers can shift functional CYP2D6 activity away from the genotype-predicted phenotype, thereby altering drug exposure, therapeutic efficacy, and toxicity. Together, these three layers ultimately influence therapeutic response, adverse drug reactions, cardiovascular disease progression, and quality of life and survival. The lower panel presents an AI-assisted precision medicine platform integrating multi-omics data, clinical and environmental information, polypharmacy and drug–gene interaction profiles, and machine-learning approaches to support individualized therapeutic recommendations and real-time adaptive clinical decision-making. Continuous monitoring and feedback are envisioned to dynamically refine risk prediction and treatment optimization. Blue, green, and purple arrows indicate genetic, pathophysiological, and pharmacological influences, respectively, whereas dashed blue arrows indicate data integration and feedback.

Collectively, this three-layer framework presents CYP2D6 as a candidate systems-level modifier whose pharmacogenetic variability, drug-metabolizing function, and potential links to redox biology may intersect in clinically relevant settings. The framework is explicitly conceptual and hypothesis-generating; it is not a validated mechanistic pathway, and the magnitude of any endogenous CYP2D6-derived redox contribution remains unknown. Its purpose is to distinguish established pharmacogenomic evidence from mechanistically plausible extrapolations, identify the most consequential evidence gaps, and define experiments to test whether CYP2D6 has biological significance beyond its established effects on drug disposition. Figure 1 summarizes the conceptual relationships among these dimensions and provides the organizational basis for the sections that follow.

## 2. CYP2D6 as a Cardiovascular Redox Modulator

### 2.1. Extrahepatic Expression of CYP2D6: Cardiovascular, Neural, and Neurovascular Dimensions

Although CYP2D6 has historically been regarded as a predominantly hepatic enzyme, its expression has also been documented in several extrahepatic tissues, particularly within the central nervous system [33,57]. This broader tissue distribution has expanded the conceptual landscape of CYP2D6 biology, raising the possibility that the enzyme exerts tissue-specific regulatory functions that operate independently of, and in addition to, its systemic pharmacokinetic role [34,35,36,37,38,39,40,41,42,43].

Within the cardiovascular system, cardiac expression of several CYP isoforms, including low levels of CYP2D6, has been reported, although CYP2D6 expression is substantially lower than that of dominant cardiac CYP isoforms [34,39,40]. However, its quantitative contribution relative to dominant cardiovascular CYP isoforms remains unclear. Importantly, much of the available human myocardial CYP expression evidence has been derived from explanted or end-stage failing hearts and from regionally sampled cardiac tissue. These limitations constrain extrapolation to healthy myocardium and preclude firm conclusions regarding the physiological abundance or cardiovascular cell-specific function of CYP2D6. Despite its relatively modest cardiac abundance, local CYP-mediated metabolism may influence myocardial redox homeostasis by generating reactive metabolites and oxidative intermediates [34,35]. Whether CYP2D6 contributes meaningfully to these processes relative to more abundant cardiac CYP isoforms remains to be established. The potential contribution of localized CYP2D6-mediated oxidative reactions to pathological myocardial remodeling or arrhythmogenic susceptibility remains speculative and has not yet been directly demonstrated in cardiovascular tissues.

In vascular endothelial cells, CYP2D6 may help regulate endothelial redox signaling and vascular tone by modulating nitric oxide (NO) bioavailability and reactive oxygen species (ROS) generation, an inference drawn from the broader vascular CYP/epoxygenase literature rather than CYP2D6-specific data [36]. Given that endothelial dysfunction is a cardinal initiating event in atherosclerosis, hypertension, and heart failure, CYP-mediated oxidative imbalance can compound endothelial inflammation and impair vasodilatory capacity [37]. Although CYP2D6 is not conventionally classified among the dominant vascular CYP isoforms, CYP-dependent arachidonic acid metabolism is an established contributor to vascular signaling and blood-pressure regulation [38].

It is important to situate this proposed CYP2D6 contribution against the dominant cardiac CYP isoform reported in the literature. Human cardiac expression studies consistently identify CYP2J2, rather than CYP2D6, as the most abundant CYP450 transcript in explanted human left ventricular samples [39]. Similarly, in human primary cardiomyocytes, CYP2J2 was reported as the dominant isozyme, with only minor contributions from CYP2D6 [40]. The principal CYP2J2/CYP2C epoxygenase pathway generates epoxyeicosatrienoic acids (EETs), which are largely cardioprotective and anti-inflammatory, in contrast to the pro-oxidative role proposed here for CYP2D6. We therefore emphasize that, within the cardiac CYP complement, CYP2D6 is a comparatively minor species; its inclusion in this review reflects its extensive genetic polymorphism and its susceptibility to inhibitor- and inflammation-driven variability, rather than a claim that it is the dominant cardiac redox-active CYP isoform.

Beyond the heart and vasculature, CYP2D6 is robustly expressed in multiple regions of the central nervous system, including the cortex, hippocampus, substantia nigra, cerebellum, and brainstem nuclei governing autonomic outflow [41]. This cerebral distribution has focused attention on CYP2D6 as a potential modulator of the brain-heart axis, the bidirectional neurohumoral network through which emotional stress, autonomic nervous system activity, and cardiovascular function are reciprocally integrated [42].

Autonomic dysregulation, particularly chronic sympathetic activation, contributes to cardiovascular disease [43]. Whether local CYP2D6 activity within neural tissues materially modifies this relationship remains to be determined.

Collectively, these observations consolidate the view that CYP2D6 functions not only as a hepatic pharmacokinetic regulator but as a distributed extrahepatic modulator of cardiovascular redox biology, neurohumoral signaling, and tissue-specific metabolic adaptation, a reconceptualization with important implications for understanding disease susceptibility and therapeutic variability in cardiovascular medicine.

### 2.2. CYP2D6 in Endogenous Neurotransmitter Metabolism and Neurohumoral Cardiovascular Regulation

Among the emerging non-pharmacokinetic functions of CYP2D6, its participation in endogenous neurotransmitter metabolism and neurohumoral cardiovascular regulation is perhaps the most conceptually significant. Unlike most CYP enzymes, which primarily process xenobiotic substrates, CYP2D6 has been implicated in metabolizing endogenous neuroactive substrates and in pathways related to dopaminergic and serotonergic signaling [44,45].

CYP2D6 has been implicated in alternative metabolic pathways involving trace amines and dopamine-related substrates, including tyramine hydroxylation [45]. However, these reactions represent auxiliary pathways and should not be interpreted as indicating that CYP2D6 is a primary enzyme governing physiological dopamine synthesis. Dopamine is an indispensable regulator of cardiovascular homeostasis, governing renal sodium handling, sympathetic neurotransmission, vascular tone, and cardiac contractility [46]. Accordingly, whether CYP2D6-dependent variation in these alternative pathways produces clinically meaningful changes in blood pressure regulation or cardiovascular autonomic function remains uncertain. Polymorphism-driven variability in CYP2D6-mediated dopaminergic flux may therefore affect blood pressure regulation and autonomic responsiveness downstream. Whether genetically determined differences in CYP2D6 activity translate into measurable differences in sympathetic activity or cardiovascular autonomic regulation remains insufficiently established.

CYP2D6 has also been implicated in the metabolism of selected serotonergic substrates [47]; however, it is not considered the principal determinant of physiological serotonin turnover. Serotonin exerts pleiotropic cardiovascular effects, including regulation of platelet aggregation, vascular tone, pulmonary vascular resistance, and autonomic outflow [57], and dysregulated serotonin signaling has been implicated in arrhythmogenesis, endothelial dysfunction, and maladaptive cardiovascular remodeling [58]. CYP2D6-dependent effects on serotonergic signaling should therefore be regarded as potentially modulatory rather than as a dominant metabolic pathway, and their direct contribution to cardiovascular vulnerability remains to be established.

The brain regions in which CYP enzymes are expressed overlap functionally with components of the central autonomic network that regulate sympathetic and parasympathetic outflow [41,59]. Whether CYP2D6 activity materially alters this autonomic balance remains a hypothesis requiring direct investigation. Pathological sympathetic predominance is a well-established feature of hypertension, heart failure, atrial fibrillation, and ischemic heart disease, and sustained autonomic imbalance is a recognized driver of adverse cardiovascular remodeling [60]. Through concurrent modulation of catecholaminergic and serotonergic pathways, autonomic imbalance affects CV disease, baroreflex sensitivity, peripheral vascular resistance, and the overall magnitude of neurohumoral stress responses [61].

Phenotype-specific effects on autonomic cardiovascular regulation have been reported in several investigations. The extent to which the CYP2D6 metabolizer phenotype alters endogenous catecholamine or trace amine concentrations sufficiently to affect cardiovascular autonomic function remains uncertain. Although CYP2D6 participates in monoaminergic metabolism, direct evidence linking PM or UM status to distinct cardiovascular autonomic phenotypes is currently limited [46,47]. Although the precise molecular mechanisms underlying these phenotype-dependent autonomic profiles remain incompletely elucidated, the convergence of pharmacogenomic and neurophysiological evidence supports a substantive role for CYP2D6 in cardiovascular neurobiology that extends beyond conventional pharmacogenomics.

These relationships may be particularly relevant under conditions of chronic psychological stress and cardiovascular disease, in which autonomic dysregulation is clinically important [62]. Collectively, the available evidence positions CYP2D6 as a molecular interface linking neurotransmitter metabolism, autonomic regulation, and cardiovascular redox homeostasis, a triad of functions whose clinical implications remain an active and compelling frontier for investigation.

### 2.3. CYP2D6-Mediated Uncoupling Reactions and Endogenous Oxidative Stress Generation

Cytochrome P450 enzymes catalyze monooxygenation reactions through highly coordinated electron-transfer processes involving NADPH-cytochrome P450 reductase and molecular oxygen [48]. Under physiological conditions, this catalytic cycle efficiently channels electrons to substrate-bound oxygen intermediates, enabling productive substrate oxidation with minimal collateral reactivity. However, experimental studies of other CYP isoforms, including CYP2B6, demonstrate that inefficient catalytic coupling can increase ROS generation through electron leakage [49]. By analogy, similar uncoupling chemistry is mechanistically plausible for CYP2D6, although direct quantitative evidence remains limited.

CYP catalytic uncoupling represents a recognized source of intracellular ROS generation [50]. Whether CYP2D6 constitutes a quantitatively important contributor to this process in cardiovascular tissues remains unknown. Substrate binding, protein conformation, and electron-transfer dynamics influence the efficiency of CYP catalytic coupling [51]. Whether specific CYP2D6 polymorphic variants exhibit systematically different ROS-generating coupling efficiencies remains incompletely defined. A particularly consequential feature of sustained CYP uncoupling is its consumption of intracellular NADPH. Because NADPH is indispensable for cellular redox buffering, excessive CYP2D6 uncoupling creates a compounding vulnerability in which ROS overproduction and antioxidant insufficiency converge to amplify oxidative injury and mitochondrial dysfunction [63].

The potential cardiovascular consequences of CYP2D6-derived ROS are multifaceted and warrant further investigation. If generated locally, CYP-derived superoxide could rapidly react with nitric oxide to form peroxynitrite, thereby potentially reducing endothelial NO bioavailability and contributing to endothelial dysfunction [64]. Concurrently, hydrogen peroxide can activate pro-inflammatory signaling cascades, induce mitochondrial membrane depolarization, and inflict oxidative damage upon proteins, lipids, and mitochondrial DNA, collectively promoting a pro-fibrotic and pro-arrhythmic intracellular milieu [65]. The magnitude of CYP2D6 uncoupling varies by phenotype and substrate. CYP polymorphisms can substantially alter catalytic function and substrate handling [66]. Whether these functional differences translate into variant-specific differences in CYP2D6 coupling efficiency and ROS generation remains unclear. In cardiovascular tissues where high metabolic demand, dense mitochondrial networks, and sustained contractile activity create inherent redox vulnerability, localized CYP2D6-derived oxidative stress may therefore disproportionately contribute to arrhythmogenesis, vascular injury, and pathological myocardial remodeling. However, direct causal evidence specifically attributing cardiovascular ROS generation to CYP2D6 remains limited.

This proposed CYP2D6 contribution should also be situated within the broader landscape of cardiovascular ROS sources. NADPH oxidases, mitochondrial electron transport chain leakage, xanthine oxidase, and uncoupled endothelial nitric oxide synthase (eNOS) are established and comparatively well-quantified sources of cardiovascular oxidative stress. By contrast, the magnitude of CYP2D6-derived ROS production, and its contribution relative to these established sources, has not been directly measured in human cardiac or vascular tissue. We therefore regard CYP2D6 as a candidate, hypothesis-generating contributor to cardiovascular redox burden rather than an established source of comparable magnitude to these canonical pathways.

**Figure 2:**
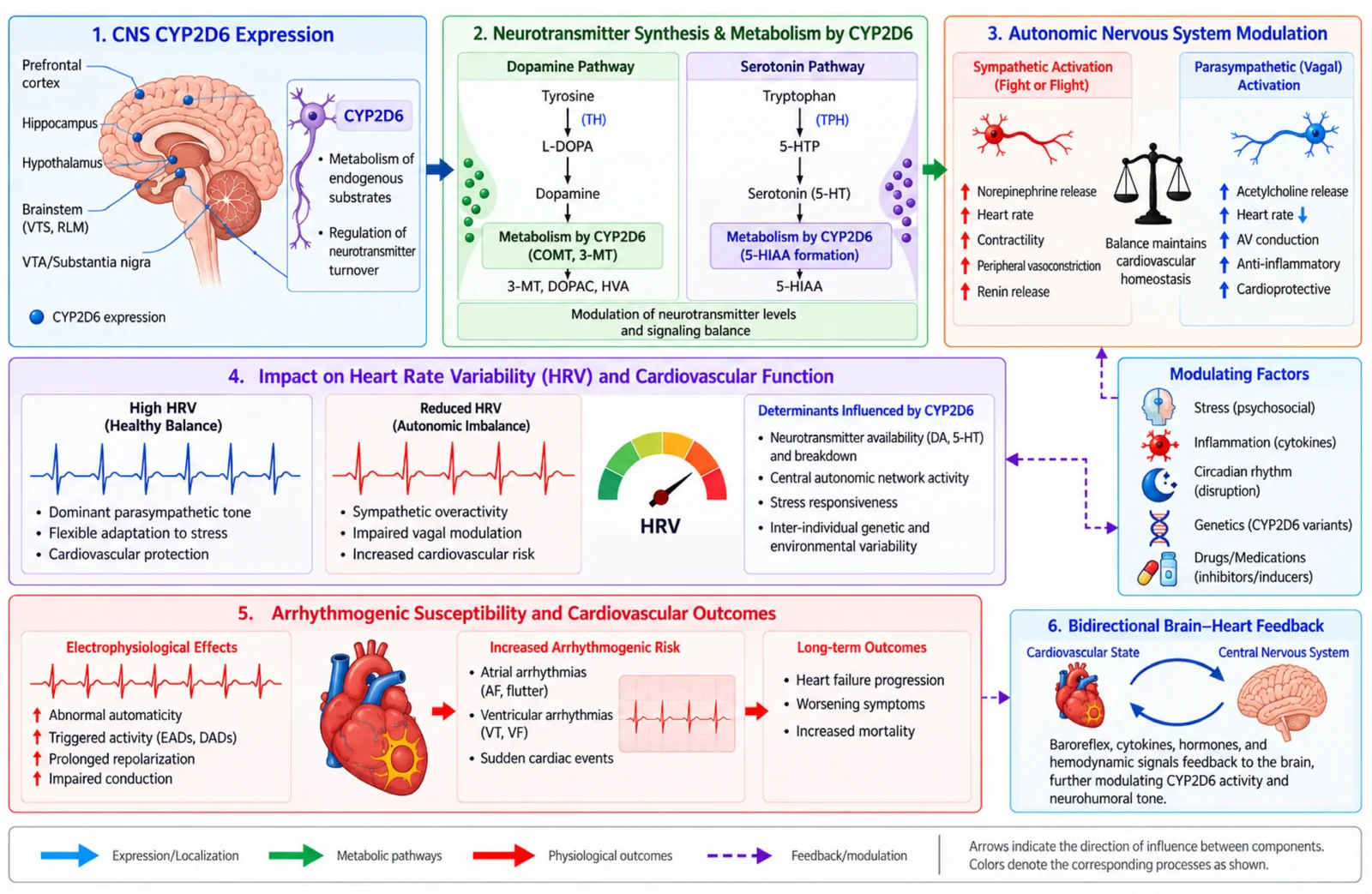
Proposed role of CYP2D6 in the brain–heart axis linking neurotransmitter metabolism, autonomic regulation, and cardiovascular function. (1) CYP2D6 is expressed in multiple regions of the central nervous system (CNS), including the cerebral cortex, hippocampus, hypothalamus, brainstem, and midbrain, where it may contribute to the metabolism of endogenous and exogenous substrates and the modulation of neurotransmitter homeostasis. (2) CYP2D6 has been implicated in pathways related to dopamine and serotonin metabolism, providing a potential mechanism through which variation in CYP2D6 activity could influence central neurotransmitter availability and signaling. (3) Changes in central neurotransmission may affect the balance between sympathetic and parasympathetic autonomic activity, thereby influencing heart rate, atrioventricular conduction, vascular tone, contractility, and other components of cardiovascular homeostasis. (4) Autonomic balance is reflected in part by heart rate variability (HRV), which is influenced by central autonomic networks, neurotransmitter signaling, stress responses, inflammation, circadian rhythms, genetic variation, and medication exposure. Altered CYP2D6 activity may therefore indirectly contribute to interindividual variability in autonomic regulation, although the magnitude and clinical relevance of this relationship remain incompletely defined. (5) Sustained autonomic imbalance, together with altered electrophysiological regulation, may increase susceptibility to atrial or ventricular arrhythmias and contribute to adverse cardiovascular outcomes in susceptible individuals. (6) Cardiovascular and central nervous system regulation are bidirectional: baroreflex signaling, circulating cytokines, neurohumoral mediators, hormones, and hemodynamic inputs provide continuous feedback between the heart and brain. Collectively, this conceptual framework illustrates how CNS CYP2D6 activity could contribute to brain–heart communication via neurotransmitter and autonomic pathways. However, several links between CYP2D6-dependent neurotransmitter metabolism, HRV, arrhythmogenesis, and long-term cardiovascular outcomes remain incompletely established mechanistically or clinically and should therefore be interpreted as proposed rather than as definitive causal relationships. The arrows indicate the direction of influence between components. Blue, green, and red arrows represent CYP2D6 expression/localization, metabolic pathways, and physiological outcomes, respectively, whereas dashed purple arrows indicate feedback or modulatory interactions.

Taken together, these findings suggest that CYP2D6 may function as more than a passive metabolic enzyme: under conditions that favor uncoupling, it potentially acts as an endogenous, phenotype-modulated source of cardiovascular oxidative stress. Importantly, the relationship between CYP2D6 activity and oxidative stress is unlikely to be linear; rather, both reduced and excessive enzymatic activity may disturb redox equilibrium through distinct mechanisms. Reduced CYP2D6 activity may promote metabolic imbalance by impairing substrate handling and altering endogenous signaling. In contrast, excessive catalytic activity may increase electron flux and favor uncoupling-mediated ROS generation under susceptible conditions. This bidirectional vulnerability highlights the need to interpret CYP2D6 phenotypes within a broader systems-level redox framework rather than a simple activity-dependent model. Figure 2 illustrates the proposed role of CYP2D6 in modulating the brain–heart axis and autonomic cardiovascular regulation.

### 2.4. Mitochondrial Dysfunction and Redox Amplification: Downstream Consequences of CYP2D6-Derived Oxidative Stress

Mitochondria serve as the principal regulators of cardiac energy metabolism and redox homeostasis. Given the exceptional energetic demands of cardiomyocytes, mitochondrial structural and functional integrity is indispensable for sustaining cardiac contractility, electrophysiological stability, and cell survival [67]. Within the hypothesis-generating framework proposed here, CYP2D6-derived ROS, if generated locally at sufficient levels, could theoretically engage mitochondrial pathways and contribute to redox amplification. Direct experimental evidence demonstrating such a CYP2D6-specific pathway in cardiovascular tissue is currently lacking.

ROS are well established to impair mitochondrial electron-transport function through oxidative damage to respiratory components, mitochondrial DNA, and membrane lipids [68]. In the framework proposed here, CYP2D6-derived ROS could theoretically contribute to this mitochondrial burden if generated locally at sufficient levels. This insult impairs oxidative phosphorylation efficiency and leads to ATP depletion, a biochemical hallmark of both failing myocardium and ischemic injury [69]. Consequent energetic insufficiency compromises ion channel activity, intracellular calcium handling, and contractile function, collectively lowering the threshold for arrhythmia and progressive cardiomyocyte dysfunction [70]. Beyond direct respiratory chain injury, an initial source of cellular ROS can trigger opening of the mitochondrial permeability transition pore, inner membrane depolarization, and secondary bursts of mitochondrial ROS release, a feed-forward phenomenon commonly referred to as ROS-induced ROS release [71,72]. This self-propagating oxidative cascade may substantially amplify the initial insult, converting a localized enzymatic source of oxidative stress into broader myocardial redox destabilization with potential for progressive cellular injury.

**Figure 3:**
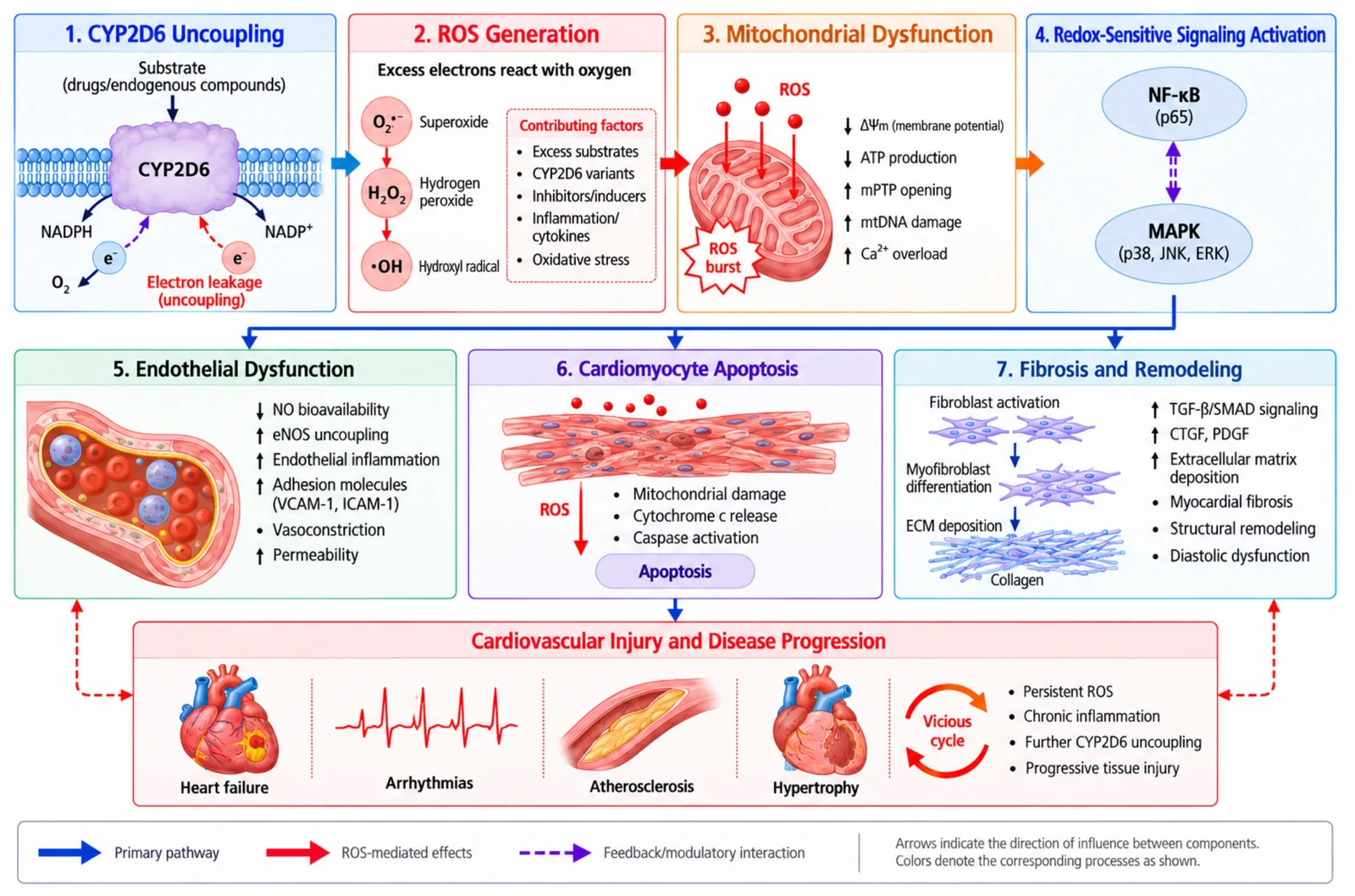
Proposed redox-mediated mechanisms linking CYP2D6 dysfunction to cardiovascular injury and disease progression. CYP2D6 catalytic uncoupling may promote electron leakage during substrate metabolism, facilitating the partial reduction of molecular oxygen and generation of reactive oxygen species (ROS), including superoxide, hydrogen peroxide, and hydroxyl radicals. Excess substrate availability, CYP2D6 genetic or functional variants, CYP2D6 inhibitors, inflammatory cytokines, and pre-existing oxidative stress may further favor redox imbalance. Increased ROS can impair mitochondrial function by reducing mitochondrial membrane potential and ATP production, and by promoting mitochondrial permeability transition, mitochondrial DNA damage, and intracellular Ca^2+^ dysregulation. Oxidative stress may also activate redox-sensitive signaling pathways, including nuclear factor-κB (NF-κB) and mitogen-activated protein kinase (MAPK) signaling. Downstream consequences include reduced nitric oxide bioavailability, endothelial nitric oxide synthase dysfunction, endothelial inflammation, increased adhesion-molecule expression, vasoconstriction, and enhanced vascular permeability. In cardiomyocytes, excessive ROS and mitochondrial injury may promote cytochrome c release, caspase activation, and apoptosis, whereas sustained oxidative and inflammatory signaling may facilitate fibroblast activation, extracellular matrix deposition, and myocardial fibrosis and remodeling. Collectively, these processes may contribute to heart failure, arrhythmias, atherosclerosis, cardiac hypertrophy, and progressive cardiovascular dysfunction. Persistent oxidative stress and chronic inflammation may further impair CYP-dependent metabolic capacity, potentially establishing a self-amplifying cycle of metabolic dysfunction, ROS generation, inflammation, and tissue injury. This model illustrates a potential mechanistic link between CYP2D6-associated redox disturbance and cardiovascular pathology; however, the strength of direct CYP2D6-specific evidence varies among individual pathways, and several proposed relationships remain to be established experimentally. Arrows indicate the direction of regulatory or signaling interactions between components. Solid arrows denote direct or primary pathway relationships, whereas dashed arrows indicate feedback or modulatory interactions. Upward (↑) and downward (↓) arrows indicate increases and decreases, respectively, in the indicated cellular or molecular parameters.

Calcium dysregulation is another major consequence of mitochondrial oxidative injury [73]. Excessive ROS impair sarcoplasmic reticulum calcium ATPases, ryanodine receptor gating, and mitochondrial calcium uniporter activity, collectively destabilizing intracellular calcium homeostasis [73]. The resulting aberrant calcium cycling promotes electrophysiological instability, may trigger arrhythmic activity, and activates apoptotic pathways in cardiomyocytes [74]. Mitochondrial dysfunction and sustained oxidative stress are firmly established drivers of heart failure progression, atrial fibrillation, diabetic cardiomyopathy, and ischemia–reperfusion injury [75]. Mitochondrial respiratory chain dysfunction is itself an established source of ROS in failing myocardium [76], providing a plausible mechanism by which an additional upstream oxidative input could be amplified. Whether CYP2D6 provides such an upstream input in vivo remains untested. These considerations position CYP2D6 as a potentially important upstream amplifier of mitochondrial dysfunction within the cardiovascular system, a role that warrants dedicated experimental scrutiny in disease-relevant cardiac models. Figure 3 summarizes the integrated consequences of CYP2D6 uncoupling, ROS amplification, mitochondrial dysfunction, inflammatory signaling, endothelial injury, and cardiac remodeling.

### 2.5. Evidence Hierarchy: Established Findings, Indirect Inference, and Critical Knowledge Gaps

To make the review’s evidentiary structure explicit, Table 1 organizes the major claims by the strength and directness of the supporting evidence. Established pharmacogenomic observations are separated from indirect mechanistic inference and from hypotheses for which direct cardiovascular CYP2D6-specific evidence is currently absent. This hierarchy is central to interpretation of the proposed framework.

## 3. Clinical Pharmacogenomics in Contemporary Cardiology

### 3.1. Evolving Pharmacogenomic Guidelines: CPIC, DPWG, and the Reassessment of CYP2D6 Activity Scores

Over the past decade, pharmacogenomic implementation guidelines for CYP2D6 have been substantially refined, driven by accumulating evidence on genotype–phenotype relationships and the clinical consequences of interindividual variability in drug metabolism. The Clinical Pharmacogenetics Implementation Consortium (CPIC) and the Dutch Pharmacogenetics Working Group (DPWG) have been instrumental in standardizing CYP2D6 phenotype classification and establishing genotype-informed prescribing frameworks [19,20,77]. Their ongoing guideline development has meaningfully advanced precision cardiology by providing clinicians with actionable pharmacogenomic decision support for cardiovascular drug selection and dose individualization.

A particularly consequential recent development is the formal reassessment of CYP2D6 activity scores (ASs) assigned to decreased-function alleles. Historically, CYP2D6 alleles were stratified using semi-quantitative scoring systems that assigned per-allele activity values ranging from 0 to 1.0, with aggregate diplotype scores used to predict phenotype class [14,78,79]. However, mounting clinical and pharmacokinetic data show that several reduced-function alleles, most notably CYP2D6*10 and CYP2D6*41, exhibit consistently lower metabolic activity than their previously assigned scores suggest [79]. In response, CPIC revised the activity score for CYP2D6*10 downward from 0.5 to 0.25, with corresponding refinements to phenotype assignment thresholds across metabolizer categories [79].

These revisions carry direct and substantial implications for cardiovascular pharmacotherapy. Reclassification of decreased-function alleles shifts population-level phenotype predictions, reassigning a meaningful proportion of individuals from normal metabolizer (NM) to intermediate metabolizer (IM) status [79,80]. Given the high prevalence of CYP2D6*10 in East Asian populations [79], this reclassification may be particularly relevant. Under updated CYP2D6 activity score criteria, some individuals previously classified as normal metabolizers may be reassigned to intermediate metabolizer status, with potential implications for the metabolism and exposure to CYP2D6-dependent drugs [79,81].

Revised CPIC and DPWG recommendations place renewed emphasis on phenotype-guided dose individualization for key cardiovascular substrates, including metoprolol, flecainide, and propafenone [82]. For poor metabolizers (PMs), dose reduction or therapeutic substitution is advised to mitigate the risk of bradycardia, conduction disturbances, and pro-arrhythmic events [82,83]. Conversely, ultra-rapid metabolizers may experience reduced exposure to CYP2D6-dependent substrates, potentially compromising therapeutic efficacy [84].

Collectively, these guideline revisions reflect a broader and necessary conceptual evolution, from static, genotype-centric interpretation toward pharmacogenomically informed, clinically contextualized precision medicine [85]. Nevertheless, genotype alone cannot fully account for variability in real-world cardiovascular drug response. This limitation is most acutely apparent in complex cardiovascular patients burdened by chronic inflammation, multimorbidity, advanced age, and extensive polypharmacy, populations in whom dynamic, non-genetic determinants of CYP2D6 activity may ultimately dominate therapeutic outcomes [86].

### 3.2. Phenotype Instability in Cardiovascular Patients: Inflammation, Comorbidities, Aging, and Frailty

Despite substantial advances in pharmacogenomic implementation, CYP2D6-guided prescribing models frequently fail to predict therapeutic outcomes with adequate precision in real-world cardiovascular practice [87]. A fundamental source of this discrepancy is the implicit assumption embedded in conventional pharmacogenomic frameworks that inherited genotype maps reliably onto a stable, enduring metabolic phenotype. In reality, CYP2D6 activity is inherently dynamic, continuously shaped by an array of physiological, pathological, and environmental determinants that can profoundly dissociate observed phenotype from genetically predicted function [88].

Chronic inflammation is among the most clinically impactful modulators of CYP2D6 activity. Elevated pro-inflammatory cytokines, including IL-6, TNF-α, and interferon-γ, suppress CYP transcription and attenuate enzymatic function through genotype-independent mechanisms [89].

Notably, CYP2D6 is classically among the more inflammation-resistant major CYP enzymes, and much of the foundational evidence for cytokine-mediated CYP suppression derives from CYP3A4, CYP1A2, and CYP2C studies rather than CYP2D6-specific data. The clinical magnitude of inflammation-driven, rather than inhibitor-driven, CYP2D6 phenoconversion in cardiovascular populations therefore warrants cautious interpretation.

Cardiovascular diseases are frequently accompanied by sustained inflammation and oxidative stress [90], providing a biological context in which hepatic CYP activity may be altered. As a consequence, genetically normal metabolizers may manifest functionally impaired drug metabolism during acute inflammatory exacerbations or chronic inflammatory disease states, a clinically silent but pharmacokinetically consequential phenotypic shift.

Chronic kidney disease (CKD) adds another layer of complexity to pharmacogenomic prediction in cardiovascular medicine. CKD is strongly associated with systemic inflammation, uremic toxin accumulation, oxidative stress, and suppression of hepatic CYP activity [91]. CKD can substantially alter nonrenal drug metabolism and hepatic CYP activity through uremic, inflammatory, and metabolic mechanisms [91,92]. Given the high co-prevalence of CKD in patients with heart failure, hypertension, and type 2 diabetes, this mechanism may substantially distort cardiovascular drug pharmacokinetics and elevate susceptibility to dose-dependent adverse events.

Aging constitutes an equally critical determinant of phenotypic instability. Elderly patients characteristically exhibit reduced hepatic perfusion, progressive mitochondrial dysfunction, chronic low-grade inflammation, sarcopenia, and altered drug distribution volumes [93]. These converging age-related changes may meaningfully alter CYP2D6 activity and amplify interindividual variability in cardiovascular drug response. Compounding this, older adults bear a disproportionate polypharmacy burden, substantially increasing the probability of drug–drug interactions and inflammation-driven phenoconversion [94].

Metabolic disorders, including type 2 diabetes mellitus and obesity, further perturb CYP2D6 regulation through intersecting mechanisms involving insulin resistance, adipose tissue inflammation, mitochondrial dysfunction, and dysregulated cytokine signaling [95]. Obesity-associated chronic inflammation may suppress CYP-mediated drug clearance, while hyperglycemia-induced oxidative stress may independently destabilize redox-sensitive metabolic pathways, compounding the risk of pharmacokinetic unpredictability [96].

Frailty represents an additional, frequently underappreciated source of pharmacogenomic complexity in cardiovascular patients. Frail individuals typically have reduced physiological reserve, impaired hepatic metabolic capacity, malnutrition-associated enzymatic insufficiency, and a heightened inflammatory burden [97]. These systemic vulnerabilities may substantially widen the gap between genotype-predicted and functionally realized CYP2D6 activity. Critically, most current pharmacogenomic algorithms fail to incorporate frailty-related physiological heterogeneity as a modifying variable, a significant oversight given the prevalence of frailty in elderly cardiovascular populations [98].

Collectively, these observations expose the inherent inadequacy of static genotype-based models when applied to the physiologically complex cardiovascular patient. They compel a transition to dynamic precision cardiology frameworks that integrate real-time inflammatory biomarkers, comorbidity burden, physiological status, and functional phenotyping to capture the full determinants of individual drug response [99].

### 3.3. β-Blockers and CYP2D6: Pharmacokinetic Vulnerability, Oxidative Risk, and Agent-Specific Considerations

β-blockers are among the most clinically consequential classes of CYP2D6-dependent cardiovascular medications. Among available agents, metoprolol is the most extensively characterized in CYP2D6 pharmacogenomics [100]. Metoprolol undergoes extensive first-pass and systemic hepatic metabolism, predominantly via CYP2D6-mediated oxidation, making plasma drug concentrations highly sensitive to CYP2D6 functional status [101].

Clinical evidence consistently shows that CYP2D6 poor metabolizers (PMs) have substantially higher metoprolol exposure than normal metabolizers (NMs), with exaggerated β-adrenergic blockade and a significantly increased risk of bradycardia, hypotension, dizziness, and atrioventricular conduction disturbances [100,101,102]. At the opposite extreme, ultra-rapid metabolizers (UMs) may achieve subtherapeutic plasma concentrations due to accelerated drug clearance, compromising antihypertensive and rate-control efficacy [103,104]. These pharmacokinetically driven divergences have informed guideline recommendations for dose reduction or therapeutic substitution in CYP2D6 PMs receiving metoprolol [79,104].

Beyond pharmacokinetic variability, emerging evidence raises concern that supratherapeutic metoprolol accumulation in CYP2D6 PMs may contribute to oxidative stress-mediated cardiotoxicity. Supratherapeutic drug exposure can, in principle, contribute to off-target mitochondrial stress [105]; however, direct evidence that CYP2D6-dependent metoprolol accumulation produces clinically meaningful oxidative cardiotoxicity remains limited. In contrast, reduced CYP2D6 activity can increase systemic exposure to CYP2D6-dependent cardiovascular drugs, potentially increasing the risk of concentration-dependent adverse effects [97,98,99,100,101,104]. Whether excessive metoprolol exposure in patients with impaired CYP2D6 activity directly contributes to mitochondrial dysfunction or redox-mediated myocardial injury remains unclear and warrants further investigation.

Carvedilol presents a pharmacologically distinct profile that confers relative resilience to CYP2D6-driven variability. Unlike metoprolol, carvedilol is biotransformed by multiple CYP isoforms, including CYP2D6, CYP2C9, and CYP3A4, distributing its metabolic liability across parallel pathways and attenuating the impact of any single-enzyme polymorphism [106]. Furthermore, carvedilol possesses intrinsic antioxidant and anti-inflammatory properties that may partially counteract ROS-mediated cardiovascular injury, adding a pharmacodynamic dimension to its favorable tolerability profile [107]. As a result, CYP2D6 polymorphisms exert comparatively modest effects on carvedilol pharmacokinetics and clinical outcomes relative to metoprolol [108].

Other clinically relevant β-blockers, including propranolol and nebivolol, exhibit varying degrees of CYP2D6 dependency with distinct pharmacogenomic implications [109]. Nebivolol is of particular interest: its combination of β_1_-selective adrenergic blockade with endothelial nitric oxide-mediated vasodilation means that CYP2D6 polymorphisms may influence not only its systemic pharmacokinetics but also its vascular pharmacodynamic responses [110]. These agent-specific differences underscore the importance of incorporating drug-level characterization of metabolic pathways, rather than class-level generalizations, when applying CYP2D6 pharmacogenomic guidance in cardiovascular practice.

### 3.4. Antiarrhythmic Agents and CYP2D6: Narrow Therapeutic Windows, Pro-Arrhythmic Risk, and Phenoconversion

Class Ic antiarrhythmic agents, principally propafenone and flecainide, represent among the most clinically consequential CYP2D6 substrates in cardiovascular medicine, owing to their narrow therapeutic indices and well-documented potential for life-threatening pro-arrhythmic toxicity [111]. In this context, CYP2D6-driven variability in drug clearance is not merely a pharmacokinetic concern but a direct determinant of electrophysiological safety, where modest deviations in plasma drug concentration can precipitate catastrophic arrhythmic events.

Propafenone undergoes extensive CYP2D6-dependent biotransformation into both active and inactive metabolites, and the parent-to-metabolite ratio is critically sensitive to metabolizer phenotype [112]. In poor metabolizers (PMs), impaired clearance results in disproportionate accumulation of the parent compound, prolonged elimination half-life, and intensified sodium channel blockade [113]. Excessive class Ic antiarrhythmic exposure can produce conduction slowing and proarrhythmic toxicity, risks that are particularly important in patients with structural heart disease [114]. Compounding this risk, propafenone possesses intrinsic β-blocking activity that may further precipitate bradycardia and atrioventricular conduction disturbances, particularly in patients with pre-existing conduction system disease or impaired left ventricular function [115].

Flecainide similarly demonstrates pronounced CYP2D6 dependency. Genotype-driven reductions in CYP2D6 activity predispose to flecainide accumulation and heightened risk of ventricular arrhythmias, with the greatest susceptibility observed in patients with structural heart disease or compromised ventricular function [116]. Because flecainide potently inhibits sodium channels in a use-dependent manner, supratherapeutic plasma concentrations can profoundly slow cardiac conduction velocity and substantially increase vulnerability to reentrant arrhythmias [117].

**Figure 4:**
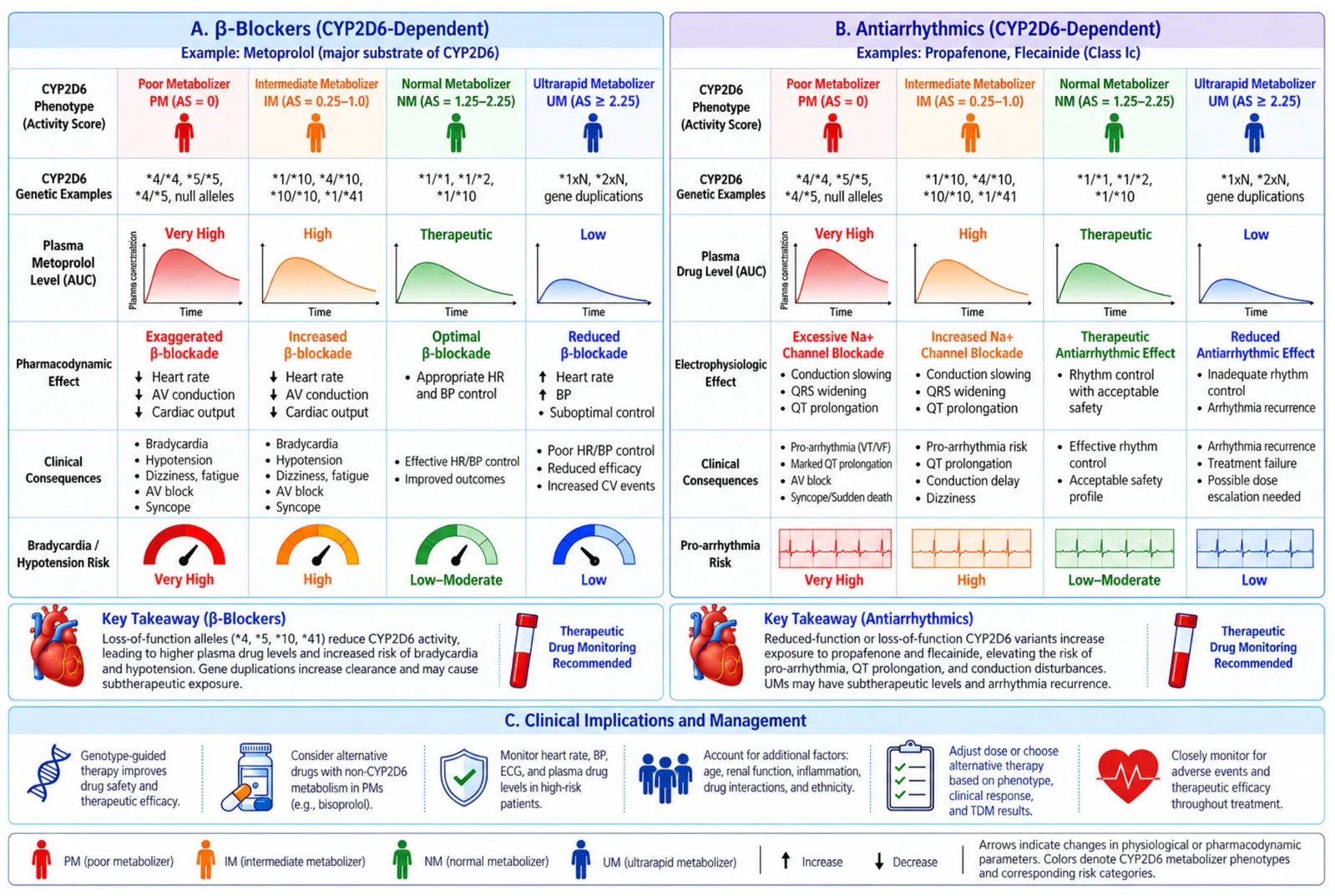
Impact of CYP2D6 metabolizer phenotype on the pharmacokinetics, pharmacodynamic responses, and cardiovascular safety of β-blockers and antiarrhythmic drugs. CYP2D6 genetic variation can substantially modify systemic exposure to selected cardiovascular drugs, resulting in phenotype-dependent differences in therapeutic response and risk of adverse effects. (**A**) Metoprolol, a CYP2D6-dependent β-blocker, illustrates the relationship between CYP2D6 metabolic capacity and drug exposure. Poor metabolizers (PMs) and intermediate metabolizers (IMs) generally exhibit reduced metabolic clearance and increased metoprolol exposure compared with normal metabolizers (NMs), potentially enhancing β-adrenergic blockade and increasing susceptibility to bradycardia, hypotension, conduction disturbances, dizziness, or syncope. Conversely, ultrarapid metabolizers (UMs) may have lower systemic exposure and potentially reduced therapeutic response. (**B**) CYP2D6-dependent Class Ic antiarrhythmic drugs, represented by propafenone and flecainide, demonstrate a similar relationship between reduced metabolic capacity and increased drug exposure. In susceptible patients, elevated exposure may enhance sodium-channel blockade and contribute to conduction slowing, QRS widening, QT-related electrophysiological abnormalities, or pro-arrhythmic risk, whereas increased metabolic capacity may reduce exposure and potentially compromise rhythm control. The magnitude and clinical consequences of these pharmacokinetic differences remain dependent on the individual drug, genotype, dose, concomitant medications, and underlying cardiovascular status. (**C**) Clinical interpretation of CYP2D6 genotype should therefore be integrated with age, renal and hepatic function, inflammation, drug–drug interactions, comorbidities, clinical response, and, where appropriate, therapeutic drug monitoring. Genotype-informed therapy may support individualized drug and dose selection, consideration of agents less dependent on CYP2D6 metabolism, and closer monitoring of heart rate, blood pressure, electrocardiographic parameters, drug exposure, therapeutic efficacy, and adverse events in high-risk patients. Collectively, these examples illustrate how CYP2D6 pharmacogenetic variability can contribute to interindividual differences in cardiovascular drug exposure and response, while emphasizing the importance of integrating genotype with dynamic clinical factors rather than using genotype alone for therapeutic decision-making. Colors distinguish CYP2D6 metabolizer phenotypes and their corresponding exposure or risk categories: red, orange, green, and blue denote PM, IM, NM, and UM phenotypes, respectively. The asterisk (*) denotes CYP2D6 star-allele nomenclature (e.g., CYP2D6*4 and CYP2D6*5) and does not indicate a footnote. Upward (↑) and downward (↓) arrows indicate increases and decreases, respectively, in the indicated pharmacodynamic or physiological parameters.

The pro-arrhythmic hazards associated with CYP2D6-dependent antiarrhythmics are amplified in high-risk clinical contexts, including elderly patients, individuals with heart failure, and those receiving concomitant CYP2D6 inhibitors [118]. Drug-induced phenoconversion, whereby a potent inhibitor such as fluoxetine, paroxetine, or quinidine functionally transforms a genetically normal metabolizer into a PM, may dramatically and unpredictably escalate antiarrhythmic toxicity risk without any change in prescribed dose [119]. This mechanism is of particular concern given the frequency with which CYP2D6 inhibitors are co-prescribed in cardiovascular patients with comorbid depression, pain, or malignancy. Genotype-guided dosing strategies and proactive therapeutic drug monitoring are therefore increasingly regarded as essential components of safe antiarrhythmic management in pharmacogenomically at-risk populations [120]. The effects of CYP2D6 variability on cardiovascular drug pharmacokinetics, therapeutic efficacy, and toxicity risk are summarized in Figure 4.

### 3.5. The CYP2D6*10 Conundrum in East Asian Populations: Allelic Prevalence, Phenotypic Reclassification, and Clinical Consequences

Among the population-specific challenges confronting CYP2D6 pharmacogenomics, the exceptionally high prevalence of the decreased-function CYP2D6*10 allele in East Asian populations stands out as one of the most clinically significant and historically underappreciated [121]. In marked contrast to European populations, where nonfunctional alleles such as CYP2D6*4 predominate as the principal driver of poor metabolizer status, CYP2D6*10 is the defining variant in East Asian pharmacogenomics, with reported allele frequencies reaching 40–60% across Chinese, Japanese, and Korean populations [5,121,122].

At the molecular level, the CYP2D6*10 variant has a Pro34Ser substitution and is associated with reduced CYP2D6 protein stability and catalytic activity [123,124]. Individuals carrying CYP2D6*10-containing diplotypes may therefore have reduced metabolic capacity, although the magnitude of this reduction is substrate-dependent and can vary with diplotype composition and other clinical modifiers [79,125]. The revised CYP2D6 activity-score framework, which reduced the activity value assigned to CYP2D6*10 from 0.5 to 0.25, has shifted the predicted phenotype classification of some CYP2D6*10-containing diplotypes toward intermediate metabolizer status [126]. Given the high prevalence of CYP2D6*10 in East Asian populations, this refinement is particularly relevant to pharmacogenomic interpretation in this population. Importantly, phenotype reclassification should not be interpreted as indicating uniformly increased cardiovascular toxicity; rather, it may have substrate-dependent implications for the metabolism and exposure of CYP2D6-dependent drugs.

**Figure 5:**
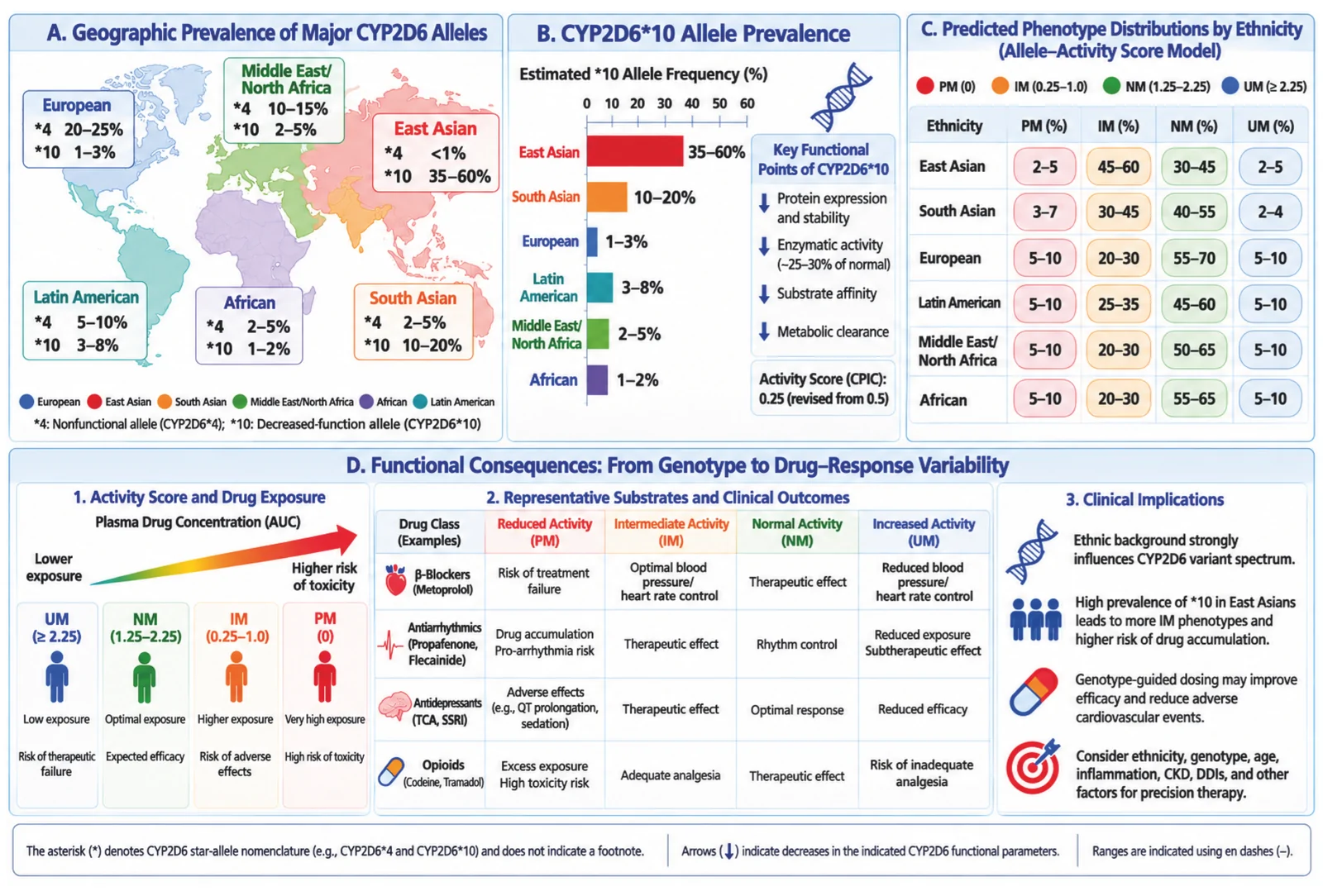
Population diversity of CYP2D6 genetic variation and its potential impact on cardiovascular drug response. (**A**) Geographic distribution of representative CYP2D6 alleles across major ancestral populations, highlighting population differences in the prevalence of the nonfunctional 4 allele and decreased-function 10 allele. (**B**) CYP2D6*10 is particularly prevalent in East Asian populations and is associated with reduced protein expression and stability, enzymatic activity, substrate affinity, and metabolic clearance. Under the revised Clinical Pharmacogenetics Implementation Consortium (CPIC) activity-score system, CYP2D6*10 is assigned an activity value of 0.25 rather than 0.5. (**C**) Representative population-level distributions of predicted poor metabolizer (PM), intermediate metabolizer (IM), normal metabolizer (NM), and ultrarapid metabolizer (UM) phenotypes illustrate how population differences in allele frequencies may influence CYP2D6 metabolic capacity. The ranges shown are approximate estimates and may vary according to cohort composition, allele coverage, and genotype-to-phenotype translation criteria. (**D**) CYP2D6 activity influences systemic drug exposure and may thereby modify therapeutic response and adverse-effect risk. Reduced metabolic activity generally increases exposure to active CYP2D6 substrates and may increase toxicity, whereas increased activity may reduce exposure and compromise therapeutic efficacy; for prodrugs requiring CYP2D6-mediated bioactivation, these effects may run in the opposite direction. Representative β-blockers, antiarrhythmics, antidepressants, and opioids illustrate the potential clinical consequences of CYP2D6-dependent variability. Importantly, ancestry or ethnicity should not be used as a surrogate for individual CYP2D6 genotype or phenotype. Precision pharmacotherapy should instead integrate genotype with age, inflammation, comorbidities, concomitant medications, drug–drug interactions, and other relevant clinical factors. The asterisk (*) denotes CYP2D6 star-allele nomenclature (e.g., CYP2D6*4 and CYP2D6*10) and does not indicate a footnote. Downward arrows (↓) indicate decreases in the indicated CYP2D6 functional parameters, including protein expression/stability, enzymatic activity, substrate affinity, and metabolic clearance.

This reclassification has direct clinical implications for cardiovascular pharmacotherapy. CYP2D6*10 carriers are at elevated risk of disproportionate drug exposure when prescribed metoprolol, propafenone, flecainide, and other CYP2D6-dependent cardiovascular agents, with corresponding increases in the risk of bradycardia, hypotension, conduction disturbances, and pro-arrhythmic toxicity [83,103,114,116,127]. These considerations expose a fundamental limitation of pharmacogenomic implementation strategies developed predominantly in European-ancestry cohorts: dosing algorithms calibrated against non-Asian allele frequency distributions may systematically misclassify East Asian patients and fail to identify individuals at elevated pharmacokinetic risk [128]. Ethnicity-informed allele interpretation and population-specific phenotype thresholds are therefore not merely refinements of existing frameworks but essential prerequisites for equitable and effective precision cardiology across genetically diverse patient populations. Figure 5 summarizes major ethnic differences in CYP2D6 allele distribution and their pharmacological implications.

### 3.6. Disease-Specific Pharmacogenomic Challenges: Heart Failure, Ischemic Disease, Atrial Fibrillation, and Cardio-Oncology

The clinical utility of CYP2D6 pharmacogenomics is not uniform across cardiovascular disease states and varies substantially with each condition’s pathophysiological milieu. Certain disease contexts introduce layers of metabolic instability that fundamentally undermine the predictive reliability of static genotype-based models, demanding disease-specific pharmacogenomic strategies.

Heart failure is frequently accompanied by hepatic congestion and altered hepatic physiology [128], while systemic inflammation and polypharmacy may further complicate drug disposition. Reduced hepatic perfusion impairs drug delivery to hepatocytes, while inflammatory cytokine signaling, operating independently of genotype, suppresses CYP transcription and enzymatic activity [129]. The net effect is functional phenoconversion in which even genetically normal metabolizers may behave pharmacokinetically as intermediate or poor metabolizers, complicating β-blocker dose titration and increasing the risk of drug accumulation and hemodynamic compromise.

Ischemic cardiovascular disease is characterized by oxidative stress and endothelial dysfunction [130], conditions that may coexist with inflammatory alterations in drug metabolism. Acute coronary syndromes are frequently accompanied by acute-phase inflammatory responses that can transiently but substantially perturb CYP activity and drug clearance at precisely the moment when pharmacokinetic precision is most critical [95]. These acute-on-chronic disruptions render genotype-guided predictions particularly unreliable during periods of active ischemic injury or post-infarction recovery.

Patients with atrial fibrillation represent a pharmacogenomically complex population by virtue of the multidrug regimens they typically require, often combining antiarrhythmics, anticoagulants, rate-control agents, and antidepressants simultaneously [131]. This polypharmacy landscape creates a high-density environment for drug–drug–gene interactions, which can alter the functional effect of CYP2D6-dependent therapies in complex cardiovascular regimens [86].

Cardio-oncology presents a distinct and increasingly prevalent source of pharmacogenomic complexity. Numerous oncology therapeutics interact directly or indirectly with CYP2D6 pathways: tamoxifen requires CYP2D6-mediated activation to generate its pharmacologically active metabolite endoxifen, while anthracyclines and tyrosine kinase inhibitors may independently amplify oxidative stress-mediated cardiotoxicity [132,133]. These interactions assume heightened clinical significance in cancer survivors with pre-existing cardiovascular disease or those receiving concurrent cardioprotective therapies, where the intersection of oncologic and cardiovascular pharmacogenomics creates bidirectional toxicity risks that neither oncology nor cardiology guidelines fully address in isolation [134].

Collectively, these disease-specific challenges expose a critical limitation of uniform, genotype-centric pharmacogenomic frameworks: the same inherited CYP2D6 genotype may yield profoundly different functional outcomes depending on the disease context in which it operates. Adaptive, multidimensional pharmacogenomic strategies that integrate genotype with disease biology, inflammatory status, comorbid burden, and real-time phenotypic assessment are therefore not a future aspiration but a present clinical necessity.

## 4. Polypharmacy, Phenoconversion, and Systems Pharmacology

### 4.1. Mechanisms of Phenoconversion: Enzyme Inhibition, Inflammatory Suppression, and Dynamic Phenotype Switching

The growing pharmacological complexity of contemporary cardiovascular medicine has exposed fundamental limitations of genotype-centered pharmacogenomic models. Although CYP2D6 genotype provides an essential baseline estimate of metabolic capacity, real-world drug metabolism often diverges substantially from genetically predicted phenotypes because of dynamic environmental, pathological, and pharmacological modifiers [135]. Phenoconversion refers to a mismatch between an individual’s genotype-predicted CYP2D6 metabolic phenotype and the functional metabolic phenotype expressed under actual clinical conditions [136,137]. In practical terms, an individual genetically classified as a normal metabolizer (NM) may function as an intermediate metabolizer (IM) or poor metabolizer (PM) when non-genetic factors reduce CYP2D6 activity, most notably concomitant administration of CYP2D6 inhibitors. Unlike inherited genotype, phenoconversion can be dynamic and reversible because functional enzyme activity may change as the responsible modifier is introduced, removed, or altered.

This concept highlights an important limitation of genotype-centered pharmacogenomic models. Although CYP2D6 genotype provides an essential baseline estimate of metabolic capacity, actual drug metabolism may deviate from the genetically predicted phenotype due to pharmacological, pathological, and environmental modifiers [135,136,137]. Among these factors, direct pharmacological inhibition provides the most established and clinically actionable mechanism of CYP2D6 phenoconversion, whereas the magnitude and clinical significance of inflammation-associated CYP2D6 phenoconversion remain less well defined.

Direct enzyme inhibition can reduce CYP2D6 activity sufficiently to shift the functional phenotype of a genetically normal metabolizer toward an intermediate- or poor-metabolizer state [138,139]. Potent CYP2D6 inhibitors, including fluoxetine, paroxetine, quinidine, and bupropion, can substantially reduce CYP2D6-mediated metabolism, thereby increasing exposure to susceptible CYP2D6 substrates [138,139]. The magnitude of this effect depends on inhibitor potency and exposure, substrate characteristics, baseline CYP2D6 activity, and concomitant medications. Because inhibitor-driven phenoconversion can emerge after treatment initiation and diminish after inhibitor withdrawal, static genotype alone may not fully predict CYP2D6 activity in polypharmacy settings.

Inflammatory suppression of CYP2D6 represents an equally important but frequently overlooked mechanism. Pro-inflammatory cytokines elaborated during chronic disease states transcriptionally downregulate hepatic CYP expression by modulating nuclear receptor signaling and downstream inflammatory transcription factors [28]. In cardiovascular patients, the convergence of chronic low-grade inflammation, oxidative stress, neurohumoral activation, and tissue hypoxia imposes a sustained suppressive burden on hepatic metabolic capacity [140]. Consequently, genotype-predicted metabolic status may substantially overestimate true enzymatic function during acute exacerbations or chronic inflammatory states, with direct implications for drug safety.

The concept of dynamic phenotype switching further underscores the temporal instability of CYP2D6 activity in clinical populations [141]. Unlike the fixed inherited genotype, actual metabolic phenotype may oscillate in response to aging, organ dysfunction, environmental exposures, circadian disruption, and evolving polypharmacy [142]. This variability is particularly pronounced in elderly patients with multimorbidity and a high medication burden, in whom a patient classified as a normal metabolizer by genotyping may transiently or persistently function as an intermediate or poor metabolizer [143].

Importantly, phenoconversion extends beyond purely pharmacokinetic effects. Emerging evidence indicates that dynamic shifts in CYP2D6 activity may reciprocally alter endogenous neurotransmitter metabolism, modulate oxidative stress generation, and perturb neurohumoral signaling. Because CYP2D6 participates in monoaminergic metabolism, changes in its functional activity could theoretically influence neurohumoral signaling; however, direct evidence linking phenoconversion to cardiovascular autonomic or redox outcomes remains limited. These observations support a systems pharmacology framework in which CYP2D6 functional output is continuously and bidirectionally shaped by the interplay among genetic architecture, inflammatory biology, oxidative stress, and the pharmacologic environment, a framework that static genotyping alone cannot adequately capture.

### 4.2. Inflammation-Induced Phenoconversion: Cytokine Mechanisms, Biomarker Correlates, and Cardiovascular Implications

Among the mechanisms contributing to CYP2D6 phenoconversion, inflammation-driven enzymatic suppression has emerged as one of the most clinically consequential in cardiovascular medicine [29]. Inflammation represents an important non-genetic contributor to phenoconversion, whereby cytokine-mediated suppression of drug-metabolizing enzymes may cause the observed metabolic phenotype to deviate from that predicted by genotype [24,144]. However, CYP2D6 appears comparatively less sensitive to inflammatory regulation than several other major CYP isoforms, and the magnitude of CYP2D6-specific suppression remains uncertain. Given the near-universal prevalence of chronic inflammation across major cardiovascular comorbidities, inflammation-mediated phenoconversion likely represents a far more common source of unpredictable drug response than current clinical practice acknowledges.

IL-6 is among the best-characterized cytokine mediators of inflammation-associated CYP transcriptional suppression [145]. Experimental studies have demonstrated cytokine-mediated suppression of CYP transcription [146], while crosstalk between inflammatory NF-κB signaling and nuclear receptor pathways provides an additional mechanistic basis for inflammation-associated alterations in xenobiotic metabolism [147]. However, direct evidence quantifying IL-6-mediated suppression of CYP2D6, particularly in individuals with genetically normal CYP2D6 function, remains limited.

This CYP2D6-specific finding notwithstanding, the broader literature on cytokine-mediated CYP suppression is derived predominantly from data on CYP3A4, CYP1A2, and CYP2C, and the clinical magnitude of inflammation-driven CYP2D6 phenoconversion remains an area of active debate.

TNF-α acts as a parallel and synergistic driver, primarily through NF-κB-mediated inflammatory transcriptional reprogramming [147,148]. Beyond transcriptional effects, TNF-α-induced oxidative stress may further destabilize mitochondrial integrity and amplify redox-mediated CYP dysregulation [31].

C-reactive protein (CRP), a readily accessible systemic inflammatory biomarker, has been associated with reduced CYP-mediated drug clearance in multiple studies, showing significant inverse correlations with CYP metabolic activity [149]. Although CRP does not directly suppress enzymatic activity, its elevation may serve as a practical surrogate marker of the inflammatory milieu driving phenoconversion. Critically, inflammation-induced CYP suppression is not static: it fluctuates dynamically with disease severity, intercurrent infection, and acute cardiovascular events, generating substantial temporal variability in drug exposure that genotype-based models cannot anticipate [150].

**Figure 6:**
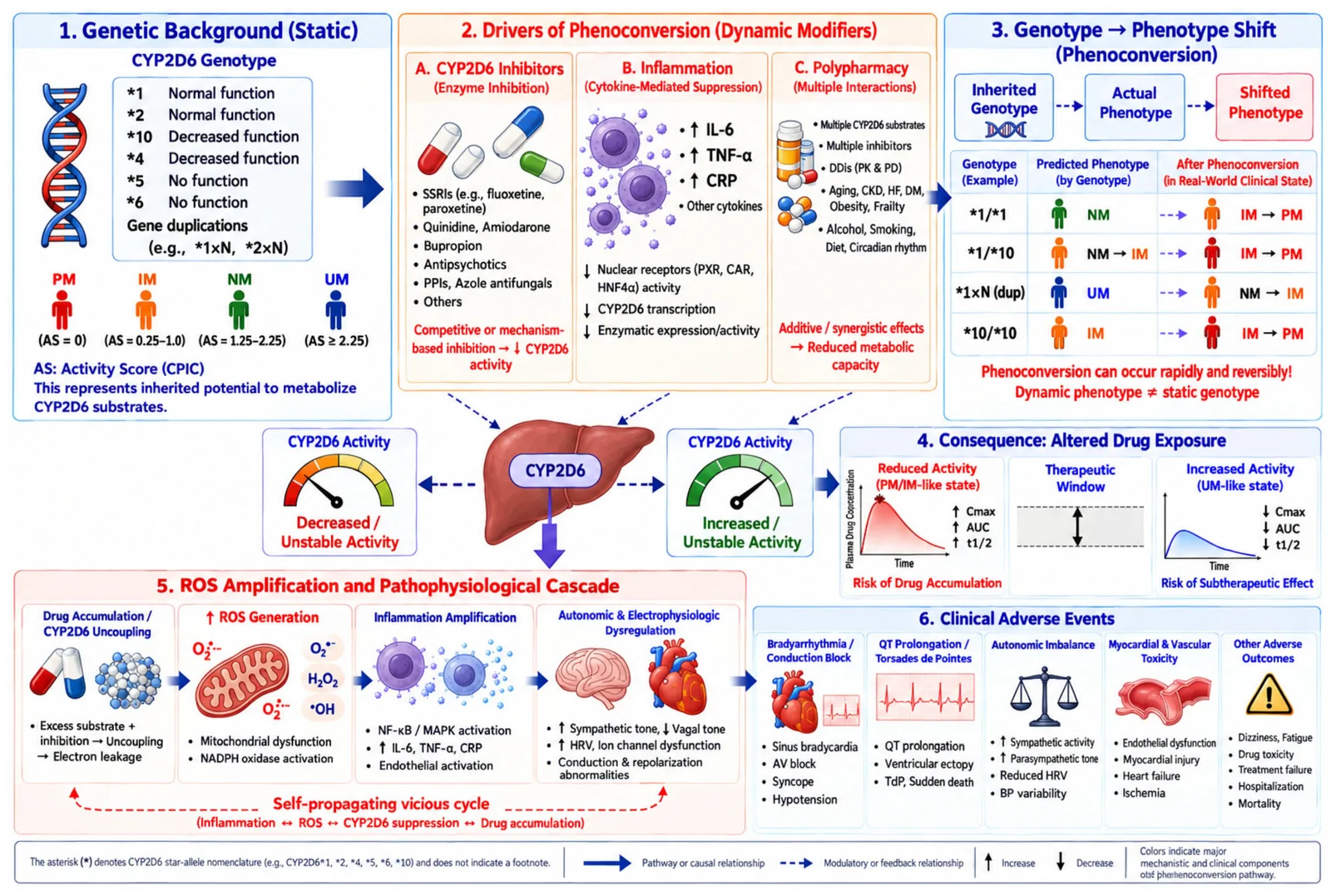
Dynamic CYP2D6 phenoconversion linking genetic background, environmental modifiers, altered drug exposure, and cardiovascular toxicity. CYP2D6 genotype establishes an inherited baseline for metabolic capacity but does not invariably predict the functional phenotype under real-world clinical conditions. CYP2D6 inhibitors, systemic inflammation, and polypharmacy can dynamically modify enzyme activity and cause phenoconversion, in which the observed metabolic phenotype deviates from the genotype-predicted phenotype. Potent CYP2D6 inhibitors may directly reduce enzymatic activity, whereas inflammatory cytokines and associated signaling pathways can suppress CYP expression and metabolic capacity. In multimorbid patients, these effects may be further influenced by multiple substrates and inhibitors, comorbidities, aging, diet, smoking, and other clinical or environmental factors. Consequently, individuals genetically classified as normal, intermediate, or ultrarapid metabolizers may transiently exhibit lower functional CYP2D6 activity, thereby altering drug exposure. Reduced CYP2D6 activity may increase systemic exposure, peak concentration, and elimination half-life of active CYP2D6 substrates, whereas increased metabolic activity may reduce drug exposure and contribute to subtherapeutic effects; the clinical consequences may differ for prodrugs requiring CYP2D6-mediated bioactivation. Excess exposure to susceptible drugs may additionally promote mitochondrial dysfunction, reactive oxygen species (ROS) generation, inflammatory signaling, endothelial injury, and autonomic or electrophysiological disturbances. Oxidative and inflammatory stress may, in turn, further impair CYP-dependent metabolic capacity, potentially establishing a self-amplifying cycle linking inflammation, oxidative stress, CYP2D6 dysfunction, and drug accumulation. These dynamic interactions may contribute to bradyarrhythmia, conduction abnormalities, QT prolongation, autonomic imbalance, myocardial or vascular toxicity, treatment failure, and other adverse clinical outcomes. Accordingly, integration of genotype with medication exposure, inflammatory and redox biomarkers, comorbidities, and longitudinal clinical monitoring may provide a more informative assessment of CYP2D6-related cardiovascular drug risk than genotype alone. The asterisk (*) is an integral part of the standardized CYP2D6 star-allele nomenclature and does not represent a footnote symbol. Arrows indicate the direction of interactions between components, whereas dashed arrows indicate modulatory or feedback relationships. Upward (↑) and downward (↓) arrows indicate increases and decreases, respectively, in the indicated parameters. Colors distinguish the major mechanistic and clinical components of the phenoconversion pathway.

These mechanisms carry direct and serious clinical implications for cardiovascular patients receiving CYP2D6-dependent therapies such as metoprolol, propafenone, and flecainide. During inflammatory exacerbations (e.g., acute decompensated heart failure or post-procedural states), transient reductions in CYP2D6 activity may lead to unexpected drug accumulation and adverse events, including bradycardia, conduction abnormalities, or arrhythmia [23,24,149,150]. Integrating inflammatory biomarkers (IL-6, TNF-α, and CRP) into adaptive pharmacogenomic frameworks is therefore a clinically urgent priority. Figure 6 illustrates the dynamic mechanisms underlying CYP2D6 phenoconversion and their downstream cardiovascular consequences.

### 4.3. Drug–Drug–Gene Interactions (DDGIs) in Cardiovascular Medicine: Cardio-Psychiatry, Cardio-Oncology, and Pain Management

#### 4.3.1. Cardio-Psychiatry Interface

Selective serotonin reuptake inhibitors (SSRIs), particularly fluoxetine and paroxetine, are clinically important CYP2D6 inhibitors and can substantially reduce CYP2D6-mediated drug metabolism [150,151]. This interaction is particularly relevant to CYP2D6-dependent cardiovascular therapies, where inhibition may increase drug exposure and alter therapeutic response or toxicity risk. A well-characterized example is the interaction between metoprolol and paroxetine or fluoxetine: concomitant administration can increase metoprolol exposure and has been associated with clinically relevant β-blocker-related adverse effects, including bradycardia and hypotension [152,153]. These interactions illustrate how pharmacological CYP2D6 inhibition can produce functional phenoconversion and increase cardiovascular drug exposure even when the inherited genotype predicts normal enzyme activity.

**Figure 7:**
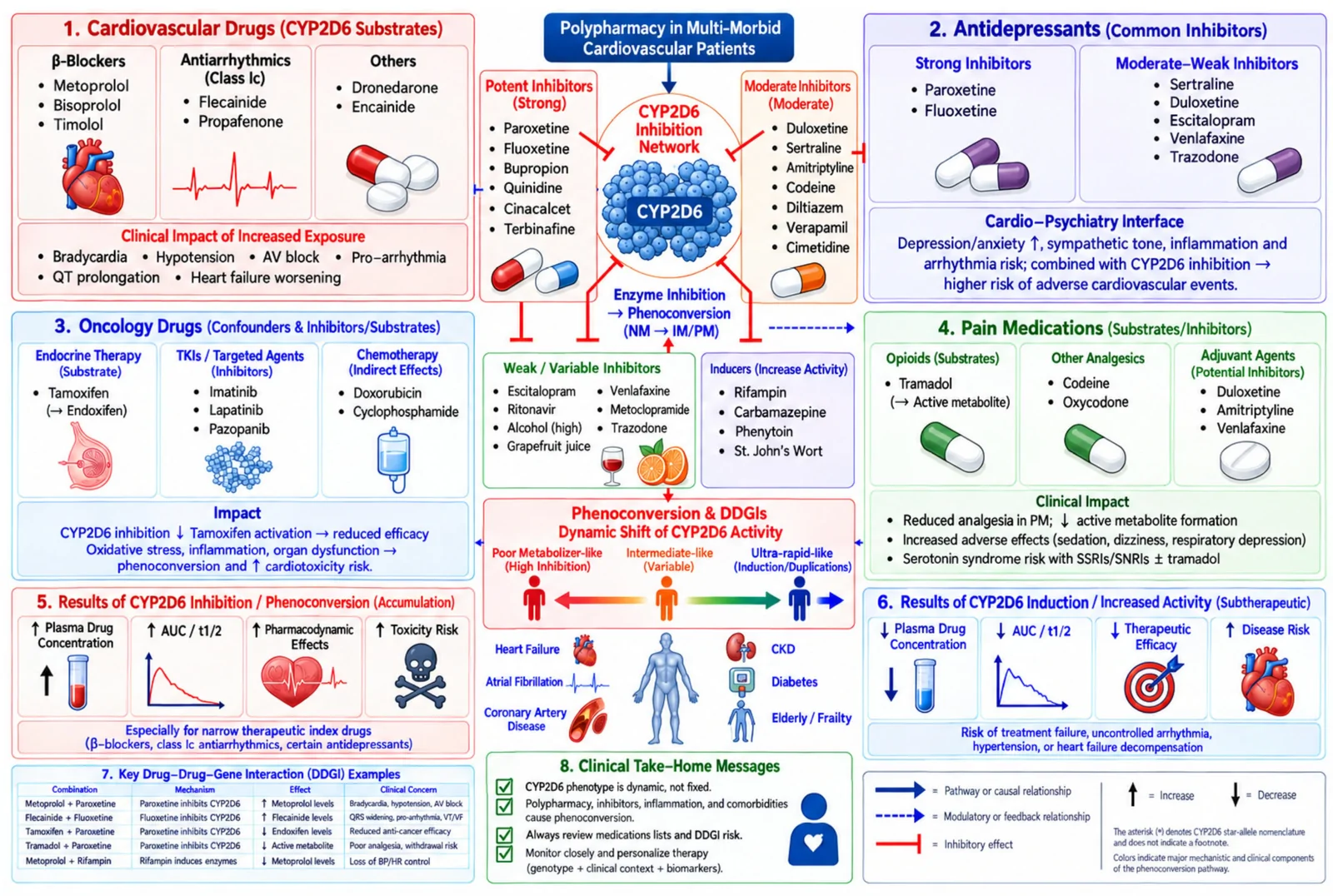
CYP2D6 phenoconversion and drug–drug–gene interactions in cardiovascular polypharmacy. Polypharmacy can dynamically modify CYP2D6 activity, thereby altering the relationship between inherited genotype and the functional metabolic phenotype. Strong or moderate CYP2D6 inhibitors may reduce enzyme activity and induce phenoconversion toward an intermediate- or poor-metabolizer-like state. In contrast, enzyme induction or increased metabolic activity may shift drug disposition toward a more rapid metabolic phenotype. These effects are particularly relevant in multimorbid cardiovascular patients receiving combinations of CYP2D6-dependent cardiovascular drugs, antidepressants, oncology therapies, and analgesics. For CYP2D6 substrates, inhibition may increase systemic drug exposure and prolong elimination, thereby increasing pharmacodynamic effects and toxicity, including bradycardia, hypotension, conduction abnormalities, pro-arrhythmia, or other drug-specific adverse effects. Conversely, increased metabolic activity may decrease exposure to active parent drugs and contribute to subtherapeutic efficacy or treatment failure. For prodrugs or agents requiring CYP2D6-dependent bioactivation, the clinical consequences may be reversed, with inhibition reducing active metabolite formation and potentially compromising therapeutic efficacy. Drug–drug–gene interactions (DDGIs) therefore arise from the combined effects of CYP2D6 genotype, inhibitors, inducers, substrate characteristics, inflammation, comorbidities, and other clinical modifiers. The magnitude and direction of these interactions are drug- and context-dependent. They may be particularly important in patients with heart failure, atrial fibrillation, coronary artery disease, chronic kidney disease, diabetes, advanced age, or frailty. Integrating genotype with medication review, clinical context, and relevant biomarkers may therefore provide a more accurate assessment of CYP2D6-related drug response than genotype alone, supporting individualized therapeutic management. Solid arrows indicate the direction of pathway or causal relationships, whereas dashed arrows indicate indirect, modulatory, or cross-panel relationships. T-bar symbols (⊣) indicate inhibitory effects. Upward (↑) and downward (↓) arrows indicate increases and decreases, respectively, in the indicated pharmacokinetic, pharmacodynamic, or clinical parameters.

Similar considerations apply to CYP2D6-dependent class Ic antiarrhythmic agents such as flecainide and propafenone. Because reduced CYP2D6 activity can increase exposure to these narrow-therapeutic-index drugs, coadministration of potent CYP2D6 inhibitors may further increase the risk of conduction disturbances and proarrhythmic toxicity in susceptible patients [113,114,115,116]. The cardio-psychiatry interface is further complicated by the cardiovascular effects of depression itself, particularly its association with autonomic dysregulation and coronary heart disease [154]. However, the extent to which depression-related inflammatory or neurohumoral changes independently modify CYP2D6 activity remains incompletely defined. Thus, cardiovascular patients receiving both CYP2D6-dependent therapies and potent CYP2D6-inhibiting antidepressants represent an important setting in which inherited genotype, pharmacological inhibition, disease-related factors, and polypharmacy may collectively determine the functional metabolic phenotype and clinical drug response. Figure 7 summarizes these complex drug–drug–gene interactions.

#### 4.3.2. Cardio-Oncology Interface

Cardio-oncology represents an increasingly important setting in which pharmacogenetic variability, polypharmacy, and treatment-related cardiovascular toxicity may intersect. Tamoxifen provides a clinically relevant example because CYP2D6 contributes significantly to its bioactivation and to the formation of the active metabolite, endoxifen. Accordingly, reduced CYP2D6 activity due to genetic variation or concomitant CYP2D6-modifying medications can decrease exposure to the active metabolite [155,156]. This interaction is particularly relevant in patients receiving antidepressants or other potent CYP2D6 inhibitors, although the clinical consequences for long-term oncologic outcomes remain incompletely defined. In patients who concurrently require CYP2D6-dependent cardiovascular medications, such polypharmacy may further complicate drug disposition and increase the potential for clinically relevant drug–drug–gene interactions.

Beyond pharmacokinetic interactions, several anticancer therapies can independently impose substantial cardiovascular stress. Anthracyclines, particularly doxorubicin, can induce mitochondrial dysfunction and oxidative injury in cardiomyocytes [157], while cardiovascular complications are recognized across multiple classes of contemporary anticancer therapies [158]. These treatment-related stresses may coexist with systemic inflammation, polypharmacy, and altered drug metabolism, creating a complex biological environment in which genetically predicted CYP2D6 activity may not fully reflect functional drug-metabolizing capacity. However, direct evidence demonstrating that anticancer therapy-induced oxidative stress or inflammation causes clinically significant CYP2D6 phenoconversion remains limited.

#### 4.3.3. Pain Management Interface

Tramadol represents a clinically relevant example of CYP2D6-dependent pharmacotherapy in patients requiring analgesic treatment. CYP2D6-mediated O-demethylation converts tramadol to O-desmethyltramadol, an active metabolite with substantially greater affinity for the μ-opioid receptor than the parent compound [159,160]. Consequently, CYP2D6 genetic variation can substantially influence exposure to the active metabolite and analgesic response, with poor metabolizers generally exhibiting reduced formation of O-desmethyltramadol and potentially diminished analgesic efficacy [160,161]. Pharmacological inhibition of CYP2D6 may similarly modify tramadol metabolism, creating genotype–phenotype discordance and further complicating prediction of therapeutic response [159,162].

An additional concern arises when tramadol is co-administered with serotonergic medications, particularly selective serotonin reuptake inhibitors, because such combinations may increase the risk of serotonin toxicity [162]. Cardiovascular manifestations of serotonin toxicity can include autonomic instability, tachycardia, and blood-pressure fluctuations, potentially complicating clinical assessment in patients with underlying cardiovascular disease. Thus, in patients receiving complex cardiovascular and neuropsychiatric pharmacotherapy, consider CYP2D6 genotype, concomitant CYP2D6 inhibitors, and serotonergic drug interactions when evaluating tramadol efficacy and tolerability.

### 4.4. Hidden Cardiovascular Toxicity of CYP2D6 Instability: Arrhythmia, Autonomic Dysfunction, and Redox-Mediated Injury

The drug–drug–gene interactions described above illustrate how inherited CYP2D6 variation, pharmacological inhibition, disease-related phenoconversion, and polypharmacy can converge to produce substantial variability in functional CYP2D6 activity. Importantly, the clinical consequences of this instability may extend beyond altered drug exposure to cardiovascular adverse effects that can be difficult to distinguish from progression of the underlying disease. Among these consequences, bradyarrhythmia and conduction disturbances represent the most directly supported manifestations of excessive exposure to CYP2D6-dependent cardiovascular drugs.

Metoprolol provides a clinically relevant example. Reduced CYP2D6 activity, whether genetically determined or due to potent enzyme inhibition, can increase metoprolol exposure and predispose susceptible patients to excessive β-adrenergic blockade, which may manifest as bradycardia, hypotension, dizziness, or syncope [100,155,156,163]. These effects may be particularly consequential in older or multimorbid patients with limited physiological reserve. Thus, an apparent deterioration in cardiovascular status may, in some circumstances, reflect altered drug disposition rather than progression of the underlying cardiac disease.

Proarrhythmic toxicity represents another clinically important concern, particularly for CYP2D6-dependent antiarrhythmic drugs with narrow therapeutic indices. Reduced CYP2D6 activity can increase exposure to agents such as flecainide and propafenone, potentially enhancing conduction slowing and proarrhythmic risk [112,113,114,115,119]. More broadly, drug-induced QT prolongation and ventricular arrhythmias are influenced by multiple interacting factors, including drug concentration, concomitant medications, electrolyte disturbances, structural heart disease, and patient-specific susceptibility [163,164]. CYP2D6 phenoconversion may therefore represent one component of a multifactorial proarrhythmic risk profile rather than an independent determinant of ventricular arrhythmia.

A less well-established dimension of CYP2D6 instability involves neurohumoral regulation. CYP2D6 participates in endogenous monoaminergic metabolism [45,47], whereas autonomic imbalance is associated with impaired heart rate variability and increased cardiovascular risk [42,61]. These observations provide a biologically plausible basis for interactions between CYP2D6 activity and brain–heart signaling. However, direct evidence demonstrating that dynamic changes in CYP2D6 activity cause clinically meaningful alterations in sympathetic–parasympathetic balance, heart-rate variability, or arrhythmia susceptibility remains limited. Accordingly, this neurohumoral pathway should presently be regarded as hypothesis-generating rather than as an established mechanism of CYP2D6-mediated cardiovascular toxicity.

A similar distinction is required for redox-mediated injury. Cytochrome P450 catalytic uncoupling is a recognized mechanism of ROS generation [48,49,50,51], while mitochondrial ROS production can amplify oxidative and inflammatory signaling in cardiovascular disease [164]. Within the framework proposed in this review, altered CYP2D6 activity could theoretically contribute an additional oxidative input when catalytic conditions favor uncoupling. However, the quantitative contribution of CYP2D6-derived ROS to cardiovascular oxidative injury remains unclear, and direct evidence linking CYP2D6 phenoconversion to mitochondrial or redox-mediated cardiovascular damage remains limited.

Taken together, these observations support a tiered interpretation of the cardiovascular consequences of CYP2D6 instability. Altered exposure to CYP2D6-dependent cardiovascular drugs and the resulting bradycardic, hypotensive, conduction-related, or proarrhythmic effects are supported by pharmacokinetic and clinical evidence. In contrast, direct effects of CYP2D6 instability on autonomic regulation and redox-mediated tissue injury remain biologically plausible but incompletely validated. This distinction is important when translating CYP2D6 pharmacogenomics into clinical practice. It provides the rationale for moving beyond genotype alone toward an integrated assessment of functional phenotype, concomitant medications, disease state, and physiological response.

### 4.5. Convergence of Polypharmacy and Redox Imbalance: A Self-Amplifying Cycle of Cardiovascular Instability

Building on the clinical and mechanistic distinctions outlined above, consider CYP2D6 instability within the broader biological context of polypharmacy, inflammation, and oxidative stress rather than as an isolated determinant of cardiovascular injury. In patients receiving multiple medications, pharmacological inhibition and disease-related phenoconversion can alter CYP2D6 activity, thereby modifying exposure to CYP2D6-dependent drugs. At the same time, cardiovascular and metabolic diseases frequently create an oxidative and inflammatory milieu that may further influence drug disposition and physiological vulnerability. The coexistence of these processes provides a plausible basis for dynamic interactions between pharmacokinetic instability and redox imbalance, although direct evidence for a self-sustaining CYP2D6-specific feedback loop remains limited.

At the molecular level, cytochrome P450 catalytic uncoupling represents a recognized source of ROS generation [67], whereas mitochondrial ROS can amplify oxidative signaling in cardiovascular disease [165]. ROS can, in turn, interact with redox-sensitive inflammatory pathways, including NF-κB signaling, thereby linking oxidative stress with inflammatory amplification [65,166]. These established mechanisms provide a biologically plausible context in which altered CYP2D6 activity could contribute to the overall cellular redox burden. However, it has not been demonstrated that drug accumulation caused by impaired CYP2D6 activity directly increases mitochondrial electron leakage or that CYP2D6-derived ROS constitutes a quantitatively important driver of this process in cardiovascular tissues.

The clinical relevance of this interaction is likely to be greatest in patients in whom several sources of vulnerability coexist. Genetic reduction or pharmacological inhibition of CYP2D6 can increase exposure to selected β-blockers and antiarrhythmic agents, with clinically relevant consequences including excessive β-blockade, bradycardia, conduction disturbances, and proarrhythmic risk [152,153,160,161,162,163]. These pharmacokinetic effects may occur against a background of cardiovascular or metabolic disease in which oxidative stress and mitochondrial dysfunction are already present, potentially reducing physiological reserve and increasing susceptibility to adverse drug responses. Importantly, however, such adverse events should be regarded as multifactorial consequences of drug exposure, disease state, comorbidities, and concomitant medications rather than as direct manifestations of CYP2D6-derived oxidative injury.

This convergence reinforces the limitations of static genotype-based prediction. CYP2D6 genotype provides an important estimate of inherited metabolic capacity. Still, the functional phenotype encountered in clinical practice may be dynamically modified by concomitant medications, inflammation, organ dysfunction, and other disease-related factors. Accordingly, precision cardiovascular pharmacotherapy requires a framework that integrates genetic information with the evolving clinical and pharmacological environment rather than interpreting genotype as a standalone predictor of therapeutic response [167]. This systems-level perspective provides a conceptual bridge from the phenoconversion and drug–drug–gene interactions discussed in this section to the emerging technologies for dynamic CYP2D6 phenotyping and precision cardiology considered in Section 5.

## 5. Translational Frontiers and Next-Generation Precision Cardiology

### 5.1. Advanced Sequencing Technologies: Resolving CYP2D6 Structural Complexity for Precision Cardiology

The growing recognition of CYP2D6 as a systems-level cardiovascular regulator has simultaneously exposed critical limitations in conventional genotyping methodologies. Traditional short-read sequencing and targeted PCR-based assays frequently fail to resolve the highly complex genomic architecture of CYP2D6, particularly in the presence of structural rearrangements, copy-number variations (CNVs), hybrid alleles, and pseudogene conversion events involving CYP2D7 and CYP2D8 [167,168,169,170,171,172]. These technical deficiencies introduce meaningful uncertainty in phenotype assignment, with consequences most severe in ethnically diverse populations carrying rare, structurally atypical, or population-specific alleles whose complexity exceeds the detection resolution of conventional platforms.

Long-read sequencing technologies, including Pacific Biosciences single-molecule real-time (SMRT) sequencing and Oxford Nanopore Technologies platforms, have emerged as transformative solutions to the structural complexity problem inherent in CYP2D6 genotyping [167]. Unlike short-read approaches, which reconstruct the sequence by assembling overlapping fragments, long-read platforms span large, contiguous genomic regions and directly phase haplotypes, enabling accurate characterization of duplicated alleles, tandem gene arrangements, hybrid gene conversions, and architecturally complex CNV configurations [167]. These capabilities are particularly valuable for resolving ultra-rapid metabolizer (UM) genotypes driven by functional gene duplications and for identifying structurally unstable alleles that may substantially perturb cardiovascular drug metabolism in ways invisible to conventional assays.

Copy number variation represents one of the most functionally consequential yet technically challenging sources of interindividual variability in CYP2D6 activity. Gene duplications and multiduplications can dramatically amplify enzymatic output and accelerate drug clearance to subtherapeutic levels, whereas gene deletions can abolish enzymatic activity [167]. Digital PCR has emerged as a methodologically superior approach to CNV quantification in this context: by partitioning DNA into thousands of discrete microreactions, digital PCR enables high-precision determination of allele copy number and substantially improves discrimination among normal, duplicated, and deleted diplotypes compared with conventional quantitative PCR methods [169,170].

Hybrid alleles arising from recombination between CYP2D6 and flanking pseudogenes introduce an additional layer of interpretive complexity [170]. Such hybrid configurations may retain partial enzymatic activity, exhibit complete loss of function, or display structural instability that renders phenotype prediction unreliable. Yet, many remain undetected or misclassified by commercial genotyping panels whose probe designs were not optimized for hybrid variant detection [171]. Integrating long-read sequencing with purpose-built bioinformatics pipelines that can resolve complex allelic architectures therefore offers a substantive pathway to improved genotype accuracy and more reliable phenotype assignment across diverse clinical populations.

Collectively, these sequencing advances signal a broader transition in cardiovascular precision medicine. Future frameworks will increasingly move beyond static structural genotyping to integrate transcriptomic, metabolomic, and real-time phenotypic data alongside genomic information, generating multidimensional, continuously updated assessments of CYP2D6 functional status and individualized cardiovascular risk that static genotyping alone cannot provide [172].

### 5.2. Multi-Omics Integration: Transcriptomics, Metabolomics, Lipidomics, Redoxomics, and Exposomics in Dynamic CYP2D6 Phenotyping

Multi-omics integration provides a complementary framework for capturing dynamic biological processes that extend beyond static genetic information and may improve systems-level characterization of cardiovascular phenotypes [173]. Within the CYP2D6 framework proposed here, integrating genomic information with transcriptomic, metabolomic, lipidomic, and redox-related data could provide a more comprehensive representation of the biological environment that influences drug metabolism and therapeutic response. Transcriptomic profiling can characterize context-dependent changes in gene expression [174,175], whereas metabolomic approaches provide functional readouts of metabolic state [176]. Lipidomic profiling may further capture cardiovascular metabolic phenotypes [177,178,179], while redox-oriented molecular profiling can provide complementary information on systemic oxidative stress and redox homeostasis [180,181]. However, CYP2D6-specific multi-omic or redox signatures that reliably reflect enzyme activity, functional phenotype, or phenoconversion remain unvalidated.

Metabolomics offers a real-time functional readout of the metabolic consequences of CYP2D6 activity states, quantifying the endogenous and exogenous small-molecule landscape that reflects integrated enzyme function across hepatic and extrahepatic compartments [176]. Given CYP2D6’s role in neurotransmitter biotransformation, xenobiotic metabolism, and oxidative intermediate generation, metabolomic profiling holds particular promise for detecting functional phenoconversion and identifying unstable metabolic states before they manifest as clinical toxicity [176]. Altered catecholamine metabolite ratios, serotonin turnover signatures, oxidized lipid intermediates, and disrupted mitochondrial substrate profiles may collectively constitute a CYP2D6-linked cardiovascular metabolic stress fingerprint with prognostic and therapeutic relevance [176].

Lipidomics has emerged as a powerful complementary tool for characterizing the cardiovascular consequences of CYP-mediated redox perturbation [177]. Oxidized phospholipids, ceramides, eicosanoids, and lipid peroxidation products are mechanistically implicated in endothelial dysfunction, atherogenesis, and heart failure progression [178]. Because CYP-mediated oxidative metabolism intersects directly with lipid signaling pathways through arachidonic acid metabolism, eicosanoid generation, and membrane phospholipid oxidation, lipidomic profiling may delineate CYP-associated cardiovascular injury signatures and provide mechanistic insight into mitochondrial membrane vulnerability that other omics layers cannot capture [179].

Redox-oriented molecular profiling may provide complementary information on systemic oxidative stress and redox homeostasis [180,181], potentially adding a functional dimension to genotype-based assessment. However, CYP2D6-specific redox signatures that reliably reflect enzyme activity, functional phenotype, or phenoconversion remain undefined.

Exposomics extends the precision cardiology framework further still by systematically capturing the full spectrum of environmental modifiers, dietary composition, smoking status, air pollutant exposure, alcohol consumption, microbiome-derived metabolites, occupational toxins, and cumulative medication burden that dynamically perturb CYP2D6 activity and contribute to phenoconversion, oxidative stress amplification, and autonomic dysregulation [182,183]. By integrating exposomic data with genomic and functional omics layers, researchers can model the full range of intrinsic and extrinsic forces that shape individual CYP2D6 activity, moving precision cardiology from static pharmacogenetic classification toward genuine, continuously adaptive systems-level cardiovascular phenotyping [184].

### 5.3. Emerging Computational Approaches for Future CYP2D6 Research

Artificial intelligence and machine-learning methods may provide useful research tools for integrating CYP2D6 genotype, medication exposure, organ function, inflammatory markers, therapeutic drug monitoring, and longitudinal physiological data [184,185,186,187,188,189]. Their value lies primarily in modeling complex interactions that static genotype-based rules struggle to capture. However, CYP2D6-specific predictive models remain in an early translational stage, and no prospective evidence shows that AI-assisted CYP2D6 modeling improves cardiovascular outcomes. Accordingly, these approaches should be viewed as exploratory platforms for hypothesis testing and model development rather than as established clinical decision-support systems.

Digital-twin concepts represent an even more prospective extension of this strategy. In principle, patient-specific computational models could integrate pharmacogenomic, pharmacokinetic, electrophysiological, and clinical data to simulate alternative treatment scenarios [190,191,192,193]. For CYP2D6, however, such applications remain conceptual because the mechanistic parameters required to model CYP2D6-specific redox effects, autonomic consequences, and cardiovascular outcomes have not been quantitatively established. Future computational work should therefore be anchored to experimentally measured CYP2D6 activity, drug exposure, and validated clinical endpoints before claims of individualized cardiovascular prediction can be justified.

**Figure 8:**
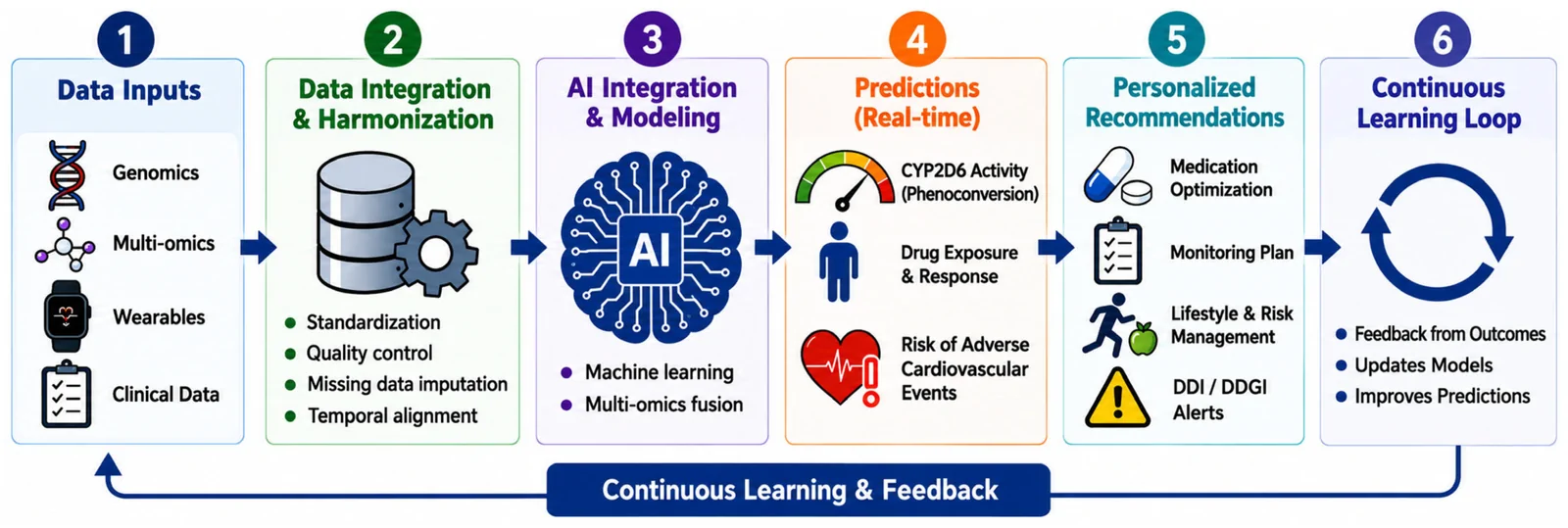
Exploratory computational framework for future integration of CYP2D6 pharmacogenomic and phenotypic data. The conceptual framework illustrates how validated genomic, pharmacokinetic, clinical, and physiological measurements could eventually be integrated by computational methods to support hypothesis testing and individualized risk modeling. The figure does not represent an established clinical decision-support system, and prospective validation would be required before any CYP2D6-specific AI or digital-twin approach could be used for clinical cardiovascular management. Colors distinguish the major stages and functional components of the proposed workflow and are used for visual organization; they do not represent quantitative values or levels of evidence.

In this review, AI and digital-twin approaches are therefore included only as future research tools that may help integrate validated pharmacogenomic and phenotypic measurements. They are not proposed as evidence for the CYP2D6–redox hypothesis itself and should not distract from the primary need for direct biochemical and physiological validation of CYP2D6-derived ROS and cardiovascular effects. Figure 8 illustrates a conceptual AI-assisted framework integrating multimodal data, real-time CYP2D6 activity prediction, personalized therapeutic recommendations, and continuous feedback.

### 5.4. Functional Phenotyping and Exploratory Physiological Monitoring

Because CYP2D6 functional activity can diverge from genotype-predicted phenotype, functional measures may complement genotyping in selected clinical and research settings. Therapeutic drug monitoring (TDM) provides the most direct clinically interpretable measure of exposure for relevant substrates. In contrast, physiological and biochemical monitoring approaches remain less specific to CYP2D6 and should be interpreted cautiously [194,195,196].

For narrow-therapeutic-index or concentration-sensitive drugs, TDM may help identify unexpected exposure during pharmacological inhibition, acute illness, or other conditions that alter drug disposition [195,196]. This use is grounded in pharmacokinetics and does not require assuming a CYP2D6-specific redox mechanism.

Wearable-derived heart rate, rhythm, and heart-rate-variability measurements can provide longitudinal information on cardiovascular physiology [197,198,199], but these signals are not CYP2D6-specific biomarkers. Their relationship to CYP2D6 functional phenotype, phenoconversion, or CYP2D6-derived ROS remains unclear. Wearables should therefore be regarded as exploratory adjuncts for future studies rather than as direct measures of CYP2D6 activity.

Similarly, circulating oxidative-stress markers such as 8-hydroxy-2′-deoxyguanosine, malondialdehyde, oxidized LDL, glutathione redox ratios, and protein carbonyls reflect integrated systemic redox biology rather than CYP2D6-specific ROS production [200,201,202]. No validated biomarker currently distinguishes CYP2D6-derived oxidative stress from ROS generated by NADPH oxidases, mitochondria, xanthine oxidase, or uncoupled eNOS. Their principal value in this context is therefore as exploratory endpoints in studies designed to test, rather than assume, a CYP2D6-specific redox contribution.

Taken together, TDM has an established role in measuring drug exposure, whereas wearables and redox biomarkers remain non-specific research tools in the CYP2D6 context. Future studies should determine whether adding these measurements to genotype and medication data improves prediction beyond conventional pharmacokinetic and clinical assessment.

### 5.5. A Dual-Track Precision Cardiology Framework: Integrating Pharmacogenomics with Dynamic Pathophysiological Monitoring

The converging insights from this review support formalizing a dual-track precision cardiology framework that moves beyond the binary choice between genotype-guided prescribing and empirical clinical judgment by integrating both into a unified, continuously adaptive therapeutic architecture [7]. In this model, inherited CYP2D6 genotype establishes the foundational layer of cardiovascular risk stratification, while real-time pathophysiological monitoring, encompassing redox biomarkers, inflammatory indices, autonomic physiology, organ function trajectories, and polypharmacy burden, provides the dynamic overlay that translates genetic predisposition into individually calibrated therapeutic action.

The first track centers on conventional pharmacogenomic profiling to characterize baseline metabolic predisposition and guide initial drug selection, dose individualization, and formulary decision-making [203]. Genotype-derived information retains essential and irreplaceable clinical value: it identifies poor and ultra-rapid metabolizer status, flags structurally complex alleles requiring advanced sequencing resolution, and delineates ethnicity-specific risk profiles, including the disproportionate CYP2D6*10 burden in East Asian populations, that must anchor the starting point of any precision therapeutic strategy.

The second track pivots from the static to the dynamic. Serial assessment of oxidative stress markers, pro-inflammatory cytokines, therapeutic drug concentrations, heart rate variability, wearable ECG parameters, and medication interaction burden provides a continuously updated functional portrait of CYP2D6 activity that genotype alone cannot supply [204]. When these multimodal data streams are integrated through AI-assisted predictive platforms that detect early signals of phenoconversion, metabolic instability, and electrophysiological vulnerability, the second track transforms from a monitoring system into a prospective intervention system, enabling preemptive therapeutic adjustments before adverse cardiovascular events materialize.

**Figure 9:**
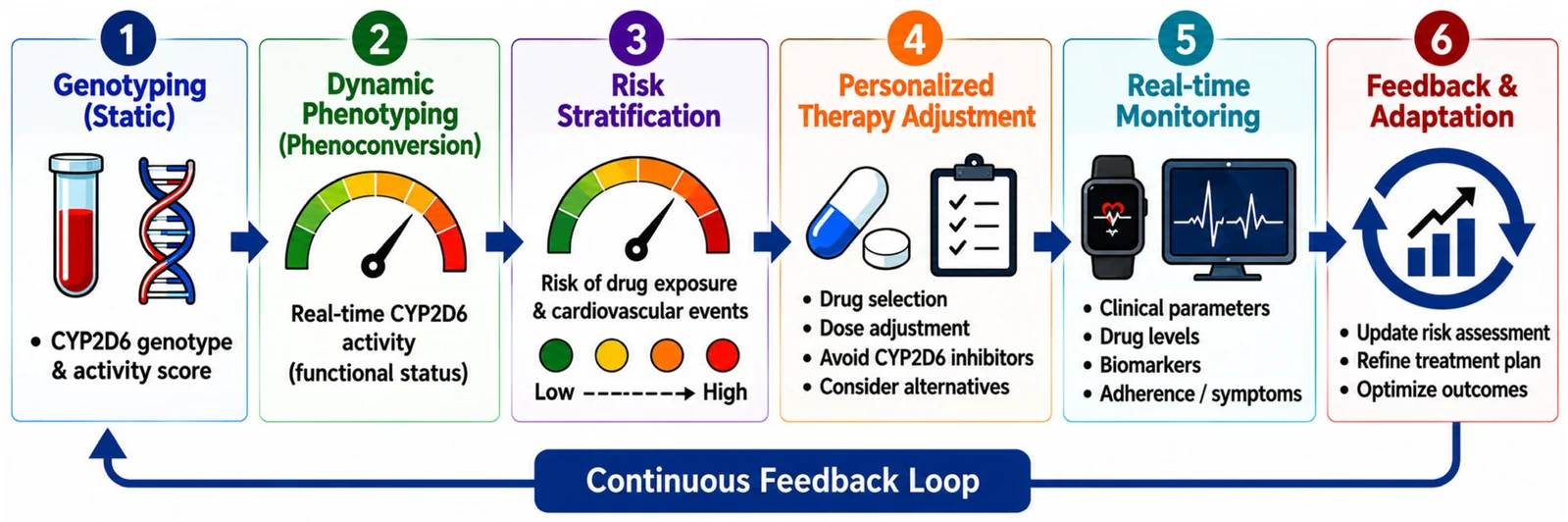
Closed-loop framework for dynamic CYP2D6-guided precision cardiovascular pharmacotherapy. The proposed framework integrates inherited CYP2D6 genotype with dynamic functional assessment, risk stratification, individualized therapeutic adjustment, and longitudinal monitoring. (1) Genotyping establishes the baseline CYP2D6 activity score and genetically predicted metabolic capacity. (2) Dynamic phenotyping incorporates real-time modifiers of CYP2D6 activity, including phenoconversion, to estimate the patient’s current functional metabolic status. (3) Genotypic and phenotypic information is integrated with relevant clinical factors to assess the risk of altered drug exposure and adverse cardiovascular events. (4) Risk assessment guides personalized therapeutic adjustment, including drug selection, dose modification, avoidance of clinically relevant CYP2D6 inhibitors, and consideration of alternative therapies. (5) Real-time monitoring of clinical parameters, drug exposure, biomarkers, treatment adherence, and symptoms enables detection of changes in therapeutic efficacy or toxicity. (6) Newly acquired clinical information is continuously fed back into the assessment process to update risk estimates, refine treatment strategies, and optimize subsequent therapeutic decisions. Collectively, this closed-loop model illustrates a transition from static genotype-based prescribing toward dynamic, phenotype-informed, and adaptive CYP2D6-guided cardiovascular pharmacotherapy. The framework is conceptual and should be prospectively validated before routine clinical implementation.

The clinical power of the dual-track framework lies in explicitly recognizing that neither track is sufficient in isolation [205]. Genotype alone fails in patients whose inflammatory disease state has converted a normal metabolizer into a functionally poor metabolizer. Dynamic monitoring without genotype lacks the foundational pharmacogenomic context needed to interpret observed variability. Together, however, they constitute a framework capable of capturing cardiovascular drug response in its full biological dimensionality, shaped by the continuous and mutually reinforcing interactions among genetic architecture, inflammatory biology, mitochondrial stress, neurohumoral signaling, and environmental exposure, and of adapting therapeutic strategy in real time as that dimensionality evolves. A proposed future clinical workflow integrating dynamic phenotyping, redox biomarkers, AI-assisted risk stratification, and adaptive therapeutic optimization is illustrated in Figure 9.

### 5.6. Barriers to Clinical Implementation: Economics, Evidence Gaps, Regulatory Challenges, and Multidisciplinary Demands

Despite the technological advances described in preceding sections, substantial and interconnected barriers continue to impede the widespread clinical implementation of CYP2D6-guided precision cardiology. Honest acknowledgment of these barriers is essential, not to diminish the paradigm’s promise, but to identify the specific obstacles that must be systematically overcome before its potential can be realized at the bedside.

Cost-effectiveness remains a primary and unresolved concern. Advanced long-read sequencing platforms, multi-omics profiling pipelines, AI computational infrastructure, and continuous physiological monitoring technologies collectively impose significant economic burdens that current healthcare reimbursement frameworks are largely unprepared to absorb [206]. Although pharmacogenomic-guided prescribing holds intuitive promise for reducing adverse drug events, avoidable hospitalizations, and downstream cardiovascular morbidity, large-scale health economic analyses with robust outcome data remain conspicuously limited, a gap that significantly constrains the evidence base needed to justify system-level investment and reimbursement policy reform.

The absence of adequately powered prospective randomized controlled trials (RCTs) represents an equally critical evidentiary limitation [207]. The preponderance of current evidence supporting CYP2D6-guided cardiovascular therapy derives from observational studies, retrospective pharmacokinetic analyses, and mechanistic investigations, study designs that, while hypothesis-generating, cannot establish definitive clinical utility or support guideline-level recommendations [208]. Large-scale RCTs specifically designed to evaluate hard cardiovascular endpoints, including mortality, arrhythmic events, hospitalization rates, and quality-adjusted survival, remain an urgent research priority if pharmacogenomically guided precision cardiology is to achieve the evidentiary standard required for mainstream clinical adoption.

Regulatory and technical standardization challenges constitute a third major implementation barrier. Substantial and clinically consequential variability persists across genotyping platforms, phenotype interpretation algorithms, CNV quantification methods, and clinical reporting formats, heterogeneity that undermines the reproducibility and cross-institutional comparability of pharmacogenomic results [209]. Before AI-assisted clinical decision support systems can be responsibly deployed at scale, they will require rigorous prospective validation, regulatory-grade performance benchmarking, and harmonization with internationally standardized pharmacogenomic reporting frameworks.

Finally, and perhaps most fundamentally, next-generation precision cardiology cannot be implemented within the boundaries of any single clinical specialty. Effective translation requires genuine multidisciplinary integration, spanning cardiologists, clinical pharmacologists, pharmacists, geneticists, bioinformaticians, data scientists, and systems biologists, operating within institutional frameworks explicitly designed to support collaborative, data-driven therapeutic decision-making [210]. Educational deficits and limited clinician familiarity with pharmacogenomic principles remain pervasive barriers to routine clinical uptake, underscoring the need for dedicated pharmacogenomics training at both undergraduate and postgraduate levels of medical education [211].

Notwithstanding these challenges, the momentum driving this field is unmistakable. The convergence of advanced sequencing technologies, multi-omics integration, AI-assisted predictive modeling, and real-time dynamic phenotyping is rapidly and irreversibly reshaping the landscape of cardiovascular precision medicine, and the barriers described here, while substantial, are implementation barriers rather than principle barriers.

## 6. Conclusions and Future Perspectives

As a narrative review, this article does not provide a systematic or quantitative synthesis of the literature. Rather, it integrates evidence from pharmacogenomics, oxidative stress biology, cardiovascular pharmacology, and precision medicine to present a plausible, clinically relevant framework for CYP2D6 in cardiovascular disease. Because direct mechanistic evidence remains incomplete in several areas, particularly regarding tissue-specific CYP2D6-derived ROS generation in cardiovascular cells, interpret the proposed model as a hypothesis-generating framework requiring further experimental and clinical validation. Accordingly, this review does not claim that CYP2D6 is already a key cardiovascular pathophysiological enzyme, but that several convergent observations justify testing this possibility. Its low abundance in cardiovascular tissue is an important counterweight to the hypothesis and makes quantitative comparison with dominant cardiac CYP isoforms and canonical ROS sources a priority for future work.

### 6.1. CYP2D6 as a Candidate Systems-Level Modifier: Synthesis and Evidentiary Boundaries

The evidence reviewed here supports a clear distinction between CYP2D6’s established pharmacogenomic importance and its proposed role in cardiovascular redox biology. CYP2D6 is a highly polymorphic drug-metabolizing enzyme with clinically relevant effects on selected cardiovascular substrates, but it is a comparatively minor cardiac CYP isoform and has not been established as a quantitatively important source of cardiovascular ROS. Its possible links to extrahepatic substrate metabolism, phenoconversion, and redox biology therefore justify focused investigation, but not classification as an established regulator of cardiovascular systems.

Extrahepatic CYP2D6 expression, particularly in the central nervous system and at low levels in cardiac tissue, provides a biological basis for investigating functions beyond hepatic drug metabolism [56]. CYP2D6 also participates in selected monoaminergic pathways; however, direct evidence that it materially alters sympathetic–parasympathetic balance, heart-rate variability, or arrhythmia susceptibility remains limited [45,47]. These observations therefore identify a plausible research interface between CYP2D6 and brain–heart signaling rather than a proven causal pathway.

The redox hypothesis requires an even stricter evidentiary distinction. Cytochrome P450 catalytic uncoupling can generate ROS [50], and oxidative stress is an established driver of mitochondrial, endothelial, inflammatory, and electrophysiological injury in cardiovascular disease [31]. However, no study has yet quantified CYP2D6-derived ROS in human cardiac or vascular tissue or established its contribution relative to major cardiovascular ROS sources such as NADPH oxidases, mitochondrial electron leakage, xanthine oxidase, and uncoupled eNOS. Consequently, CYP2D6 should presently be regarded as a candidate, hypothesis-generating redox modifier rather than an established upstream driver of cardiovascular oxidative injury.

The strongest immediate clinical relevance of CYP2D6 remains pharmacogenomic: inherited variation, inhibitor-driven phenoconversion, and polypharmacy can alter exposure to CYP2D6-dependent drugs [212]. Whether CYP2D6 also exerts clinically meaningful endogenous redox or neurohumoral effects is a separate question that requires direct experimental validation. Maintaining this distinction is essential to avoid conflating established pharmacokinetic effects with proposed pathophysiological mechanisms.

### 6.2. Integrating Genetics, Redox Biology, and Polypharmacy: Toward a Unified Systems Framework for Cardiovascular Precision Medicine

A central and recurring theme throughout this review is the fundamental inadequacy of pharmacogenomics, in isolation, as a framework for cardiovascular precision medicine. Inherited genotype establishes the biological foundation of CYP2D6 metabolic capacity, but real-world cardiovascular drug response is ultimately determined by the dynamic intersection of genetics, inflammation, oxidative stress, organ dysfunction, and polypharmacy, forces that interact continuously, nonlinearly, and in ways that no static genotype-based model can anticipate [213].

Phenoconversion stands as the most clinically compelling demonstration of this inadequacy. Patients genomically classified as normal metabolizers may transiently or persistently acquire the pharmacokinetic profile of intermediate or poor metabolizers in response to inflammatory cytokine activation or pharmacological CYP2D6 inhibition, a transformation that occurs silently, without any change in the genotype that informed the original prescribing decision [214]. The cardiovascular consequences of phenoconversion-driven drug accumulation may include bradycardia, conduction disturbances, hypotension, and pro-arrhythmic toxicity [100,163,164,165]. Mitochondrial ROS can participate in self-amplifying oxidative signaling [166]; whether CYP2D6 instability constitutes a clinically relevant upstream trigger of this process remains to be established.

Polypharmacy adds substantial complexity because drug–drug–gene interactions can reshape CYP2D6 functional activity and alter exposure to individual substrates. These pharmacokinetic effects are clinically relevant and may coexist with inflammation, oxidative stress, and organ dysfunction; however, the contribution of CYP2D6-specific redox amplification to this combined risk remains unquantified. The principal established concern is therefore genotype–phenotype discordance and altered drug exposure, whereas redox amplification remains a mechanistic hypothesis requiring direct validation.

Future precision approaches should first build on validated components—genotype, medication history, organ function, clinical phenotype, and where appropriate therapeutic drug monitoring—before incorporating exploratory redox, wearable, or computational measures. Multidimensional integration may ultimately improve prediction, but its incremental value and clinical utility must be demonstrated prospectively rather than assumed.

### 6.3. Research Priorities and Future Directions

Testing the proposed framework requires a sequence of evidence-building studies in which direct mechanistic validation precedes broad translational implementation.

The highest-priority questions are the following: (1) Can CYP2D6-derived ROS be directly and quantitatively measured in human cardiac and vascular cells or tissues? (2) What fraction of total cardiovascular ROS, if any, is attributable to CYP2D6 relative to NADPH oxidases, mitochondrial electron leakage, xanthine oxidase, and uncoupled eNOS? (3) How do cardiovascular CYP2D6 abundance and catalytic activity compare quantitatively with CYP2J2 and with hepatic CYP2D6? (4) Do CYP2D6 genotype or functional variants measurably alter ROS-generating coupling efficiency under physiologically relevant substrate concentrations? (5) What is the independent clinical magnitude of inhibitor-driven versus inflammation-associated phenoconversion? and (6) Do CYP2D6-guided interventions improve hard cardiovascular outcomes in prospective trials? These questions define the evidence sequence needed to determine whether the proposed redox component is physiologically significant.

Mechanistic studies should therefore precede large-scale systems integration. Recombinant-enzyme experiments, human cardiomyocytes and vascular cells, CYP2D6 gain- and loss-of-function models, isoform-selective inhibition, and direct ROS flux measurements should establish causality and quantify effect size. Comparative experiments should include CYP2J2 and canonical cardiovascular ROS sources so that any CYP2D6 signal can be interpreted in physiological context. Multi-omics approaches may then help identify downstream signatures, but such signatures should not be considered CYP2D6-specific unless anchored to direct enzyme-level measurements.

Prospective clinical studies should focus first on established pharmacogenomic questions, including drug exposure, concentration-dependent adverse effects, and inhibitor-driven phenoconversion. Well-powered trials are still needed to determine whether CYP2D6-guided strategies improve major arrhythmic events, cardiovascular hospitalizations, or mortality. Exploratory biomarkers, wearable signals, and inflammatory measures may be incorporated as secondary mechanistic endpoints, provided they are not interpreted as direct surrogates of CYP2D6-derived ROS without validation.

Only after establishing these mechanistic and clinical foundations should AI, machine learning, or digital twin approaches be used to integrate multidimensional CYP2D6 data [184,187,188,189,190,191,192,193,194,215]. Their appropriate near-term role is hypothesis generation, data integration, and prospective model testing, not autonomous clinical decision-making. Any model intended for clinical use should be trained on directly measured CYP2D6 function and externally validated against pharmacokinetic and cardiovascular outcomes.

Additional priorities include improved long-read sequencing for structural CYP2D6 variation, standardized functional phenotyping, and multicenter collaboration across cardiovascular pharmacology, genetics, redox biology, and quantitative physiology. Progress should be judged by whether these efforts close the central evidence gaps identified above, particularly the absence of direct quantitative evidence for a physiologically meaningful CYP2D6-derived cardiovascular ROS signal.

### 6.4. Final Perspective

CYP2D6 is firmly established as a highly variable pharmacogenetic determinant of drug disposition, but its proposed endogenous role in cardiovascular redox biology remains an open question. Current evidence supports investigating CYP2D6 as a candidate modifier because pharmacogenetic variability, clinically important cardiovascular substrates, phenoconversion, extrahepatic expression, and general CYP uncoupling chemistry converge on a testable hypothesis. That convergence, however, does not establish physiological significance.

The most important unresolved issue is quantitative. At present, there are no comparative data demonstrating that CYP2D6-derived ROS contributes meaningfully to total ROS production in human cardiac or vascular tissue relative to NADPH oxidases, mitochondrial electron leakage, xanthine oxidase, or uncoupled eNOS. Until such evidence is available, interpret the CYP2D6–redox component of this review as a hypothesis-generating framework rather than an established mechanism of cardiovascular disease.

Accordingly, the immediate clinical relevance of CYP2D6 lies primarily in pharmacogenomics, drug exposure, and phenoconversion. Multi-omics, wearable monitoring, AI, and digital-twin approaches may become useful research tools for integrating validated measurements, but they remain prospective and should not be used to infer CYP2D6-specific redox effects or guide cardiovascular care without prospective validation. The value of the framework proposed here is therefore not that it establishes CYP2D6 as a cardiovascular ROS driver, but that it defines a falsifiable set of mechanistic and clinical questions that can determine whether such a role exists and, if so, whether it is physiologically and clinically meaningful.

## Figures and Tables

**Table 1:** Evidence hierarchy for the proposed role of CYP2D6 in cardiovascular pharmacogenomics and redox biology.

Evidence Domain	Current Evidence Level	Interpretation/Key Remaining Gap
CYP2D6 genetic variation and drug metabolism	Direct/well established	CYP2D6 star alleles and copy-number variation alter baseline metabolic capacity, and CYP2D6 contributes importantly to exposure and response for selected cardiovascular substrates, including metoprolol, flecainide, and propafenone [12,17,18].
Inhibitor-driven phenoconversion and drug–drug–gene interactions	Direct/clinically supported	Potent CYP2D6 inhibitors can reduce functional enzyme activity and create genotype–phenotype discordance [23,24,26]. The magnitude of inflammation-driven CYP2D6 phenoconversion is less certain and should not be treated as equivalent to inhibitor-driven phenoconversion [28,29].
Extrahepatic CYP2D6 expression and endogenous substrate metabolism	Direct expression evidence; physiological significance uncertain	CYP2D6 is expressed extrahepatically, particularly in the CNS and at comparatively low levels in cardiac tissue, and participates in selected monoaminergic pathways. Whether this activity materially modifies cardiovascular autonomic physiology remains unproven [38,39,40,41,42,43,44,45,46,47,48,49,50,51,52,53,54,55,56].
CYP2D6 catalytic uncoupling as a cardiovascular ROS source	Indirect mechanistic inference/very low direct cardiovascular evidence	ROS generation through CYP catalytic uncoupling is established for the P450 system and has been demonstrated for other CYP isoforms [48,49,50,51]. However, CYP2D6-derived ROS has not been quantitatively established in human cardiac or vascular tissue, and no comparative data define its contribution relative to NADPH oxidases, mitochondrial electron leakage, xanthine oxidase, or uncoupled eNOS.
AI, multi-omics, digital twins, and wearable-assisted CYP2D6 phenotyping	Exploratory/prospective	These approaches may eventually integrate genotype, drug exposure, physiological monitoring, and clinical modifiers, but no prospective evidence currently shows that CYP2D6-specific AI, digital-twin, multi-omic, or wearable strategies improve hard cardiovascular outcomes. They should therefore be regarded as research tools and future hypotheses rather than validated clinical systems.

## Data Availability

No new data were created or analyzed in this study. Data sharing is not applicable to this article.

## Abbreviations

8-OHdG, 8-hydroxy-2′-deoxyguanosine; AI, artificial intelligence; AS, activity score; ATP, adenosine triphosphate; AUC, area under the concentration–time curve; AV, atrioventricular; BP, blood pressure; CAR, constitutive androstane receptor; Ca^2+^, calcium ion; CKD, chronic kidney disease; Cmax, maximum plasma concentration; CNV, copy-number variation; CPIC, Clinical Pharmacogenetics Implementation Consortium; CRP, C-reactive protein; CTGF, connective tissue growth factor; CV, cardiovascular; CYP2D6, cytochrome P450 2D6; DDI, drug–drug interaction; DDGI, drug–drug–gene interaction; DGI, drug–gene interaction; DM, diabetes mellitus; ECG, electrocardiography; ECM, extracellular matrix; eNOS, endothelial nitric oxide synthase; ERK, extracellular signal-regulated kinase; GSH/GSSG, reduced/oxidized glutathione ratio; HF, heart failure; HR, heart rate; HRV, heart rate variability; hs-CRP, high-sensitivity C-reactive protein; H_2_O_2_, hydrogen peroxide; ICAM-1, intercellular adhesion molecule-1; IL-6, interleukin-6; IM, intermediate metabolizer; JNK, c-Jun N-terminal kinase; LDL, low-density lipoprotein; MAPK, mitogen-activated protein kinase; MDA, malondialdehyde; mPTP, mitochondrial permeability transition pore; mtDNA, mitochondrial DNA; NADPH, reduced nicotinamide adenine dinucleotide phosphate; NF-κB, nuclear factor-κB; NGS, next-generation sequencing; NM, normal metabolizer; NO, nitric oxide; O_2_•^−^, superoxide anion; PM, poor metabolizer; PPI, proton pump inhibitor; PXR, pregnane X receptor; QRS, QRS complex; QT, QT interval; QTc, corrected QT interval; ROS, reactive oxygen species; SNP, single-nucleotide polymorphism; SNRI, serotonin–norepinephrine reuptake inhibitor; SpO_2_, peripheral oxygen saturation; SSRI, selective serotonin reuptake inhibitor; t_1_/_2_, elimination half-life; TDM, therapeutic drug monitoring; TdP, torsades de pointes; TGF-β, transforming growth factor-β; TKI, tyrosine kinase inhibitor; TNF-α, tumor necrosis factor-α; UM, ultrarapid metabolizer; VCAM-1, vascular cell adhesion molecule-1; VT/VF, ventricular tachycardia/ventricular fibrillation; ΔΨm, mitochondrial membrane potential; •OH, hydroxyl radical.
